# Supplementary material for: An essential role for tungsten in the ecology and evolution of a previously uncultivated lineage of anaerobic, thermophilic Archaea
Source: Nat Commun. 2022 Jun 30;13:3773. doi: 10.1038/s41467-022-31452-8 (PMC9246946; doi:10.1038/s41467-022-31452-8)
Supplement: Supplementary file 1 — Supplementary Information [file 41467_2022_31452_MOESM1_ESM.docx]

*Supplementary Information*

*for*

**An essential role for tungsten in the ecology and evolution of a previously uncultivated lineage of anaerobic, thermophilic Archaea**

Steffen Buessecker^1^*, Marike Palmer^1^*, Dengxun Lai, Joshua Dimapilis, Xavier Mayali, Damon Mosier, Jian-Yu Jiao, Daniel R. Colman, Lisa M. Keller, Emily St. John, Michelle Miranda, Cristina Gonzalez, Lizett Gonzalez, Christian Sam, Christopher Villa, Madeline Zhuo, Nicholas Bodman, Fernando Robles, Eric S. Boyd, Alysia D. Cox, Brian St. Clair, Zheng-Shuang Hua, Wen-Jun Li, Anna-Louise Reysenbach, Matthew B. Stott, Peter K. Weber, Jennifer Pett-Ridge, Anne E. Dekas, Brian P. Hedlund, Jeremy A. Dodsworth*

^1^ These authors contributed equally.

* Correspondence to: S. Buessecker (sbuessecker@stanford.edu), M. Palmer (marike.palmer@unlv.edu), J.A. Dodsworth (JDodsworth@csusb.edu)

**Supplementary Tables and Figures**  **Page/File**

**Table 1.** Media conditions of anaerobic cultures 1

**Table 2.** RT-PCR primer sequences 2

**Table 3.** CARD-FISH probe sequences tested 3

**Figure 1.** Cultivation Schematic 4

**Figure 2.** Growth experiment testing for media composition and substrate 5

**Figure 3.** Effects of tungsten on DNA concentrations 6

**Figure 4.** ASV abundances for 16S rRNA gene tags from synthetic medium 7

**Figure 5.** RT-PCR results 8

**Figure 6.** ^13^C and ^15^N enrichments in FISH(+) and FISH(–) cells 9

**Figure 7.** Alternate phylogeny for the tungsten-dependent oxidoreductases 10

**Figure 8.** Summary of tungsten-dependent oxidoreductase lineages 11

**Figure 9.** Sequence homologs of the Mbh complex 12

**Figure 10.** Archaeal phylogeny placing *Wolframiiraptoraceae* phylogenetically 13

**Figure 11.** ASR and presence/absence of genes in the family 15

**Figure 12.** Phylogeny for A subunits of Mo and W transporters 17

**Figure 13.** Relative *modA*, *tupA*, and *wtpA* gene frequencies in metagenomes 19

**Figure 14.** Relative *modA* and *tupA* gene frequencies with molar concentration 21

**Supplementary Notes**

Supplementary Note 1 22 Supplementary Note 2 23

Supplementary Note 3 24

Supplementary Note 4 26

Supplementary Note 5 27

Supplementary Note 6 33

**Supplementary Datasets**

Trace metal data for GBS spring water Data 1

Tungsten levels in GBS spring water and culture media Data 2

Information on genomes used in the study Data 3

Average Amino Acid and Nucleotide Identities Data 4

GraftM results summary Data 5

COUNT session file for ancestral character state reconstruction Data 6

Tungsten-dependent ferredoxin oxidoreductase phylogeny Data 7

Single-gene phylogenies for physiology-related genes Data 8

Normalized ASV counts Data 9

**Supplementary Table 1. Media conditions of anaerobic cultures**. Synthetic medium was GBS salts medium^53^. All cultures were grown in pure N_2_ headspace with 0.02% cysteine and 0.02% sodium sulfide. All cultures also had a mix of vitamins/yeast extract/phosphate added (see main text). Note that A6 and A6H had identical medium composition but were incubated at two different temperatures.

* Culture condition used for long term maintenance and for experiments with tungsten.

** Corn stover in A7 was autoclaved in 50 mL of synthetic medium (1 g in a nylon mesh bag) to remove easily solubilized compounds and subsequently dried before addition to media.


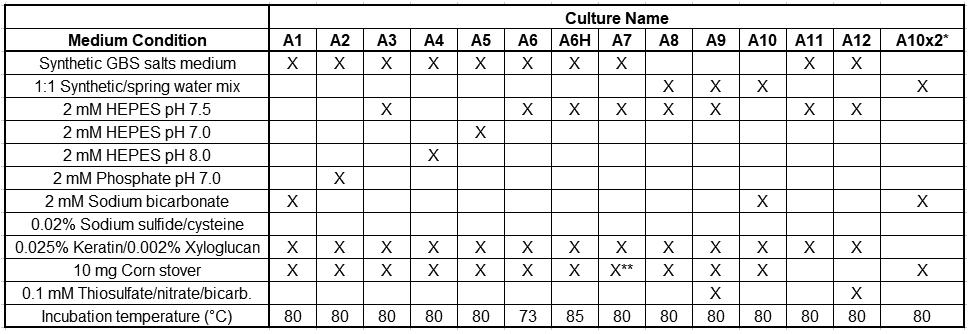


**Supplementary Table 2.** **Primers for qPCR targeting 16S rRNA genes.**

| **Target** | **Primer name** | **Sequence (5’ to 3’)** | **Annealing temperature (°C)** |  |
| --- | --- | --- | --- | --- |
| AigG4 16S rRNA gene | AigG4_f* | [Cy3]GGAGCATCCCCGTRCACCAC | 64 |  |
| AigG4 16S rRNA gene | AigG4_r* | [Cy3]AAAAGCCGGCACGAGGCC | 64 |  |
| Arch/Bact 16S rRNA genes | 515uFmod | GTGYCAGCMGCCGCGGTAA | 55 |  |
| Arch/Bact 16S rRNA genes | 806r | GGACTACHVGGGTWTCTAAT | 55 |  |
|  |  |  |  |  |

* These AigG4-specific primers were also used in RT-PCR

**Supplementary Table 3. RT-PCR primer sequences.**

| **Target** | **ID** | **NCBI Protein accession** | **Sequence name** | **Sequence (5’ to 3’)** | **Annealing temperature (°C)** |
| --- | --- | --- | --- | --- | --- |
| Tungstate ABC transporter, permease portion | peg232 | MCF3653658.1 | ABC1_f | CCTCAAGATAGCGGCCCTAA | 63.1 |
| Tungstate ABC transporter, permease portion | peg232 | MCF3653658.1 | ABC1_r | CTTTCATGGCCGCATCTTCC | 63.1 |
| Tungstate ABC transporter, tungstate-binding portion | peg233 | MCF3653659.1 | ABC2_f | ACCTACTCTCATTCAGGCGG | 63.1 |
| Tungstate ABC transporter, tungstate-binding portion | peg233 | MCF3653659.1 | ABC2_r | ATTATGGCGTGGGGTGTGTA | 63.1 |
| Tungstate ABC transporter, ATP-binding portion | peg234 | MCF3653660.1 | ABC3_f | CTGATCCGGCAAAAATCAAT | 61.4 |
| Tungstate ABC transporter, ATP-binding portion | peg234 | MCF3653660.1 | ABC3_r | TGTCTGCTCCATGCCTACAG | 61.4 |
| Tungsten-containing aldehyde:ferredoxin oxidoreductase (EC 1.2.7.5) | peg370 | MCF3653440.1 | AOR1_f | TGCGGTCTTCAAGTCTCCTT | 61.4 |
| Tungsten-containing aldehyde:ferredoxin oxidoreductase (EC 1.2.7.5) | peg370 | MCF3653440.1 | AOR1_r | TCCCGGGGTAATCTCTCTGA | 61.4 |
| Tungsten-containing aldehyde:ferredoxin oxidoreductase (EC 1.2.7.5) | peg692 | MCF3653608.1 | AOR2_f | ATATGATGAGGAGGGCTGGC | 61.4 |
| Tungsten-containing aldehyde:ferredoxin oxidoreductase (EC 1.2.7.5) | peg692 | MCF3653608.1 | AOR2_r | TCGAGGCATAGACCACCATC | 61.4 |
| Tungsten-containing aldehyde:ferredoxin oxidoreductase (EC 1.2.7.5) | peg1086 | MCF3653595.1 | AOR3_f | ACCAGCATATGACCCGAGAG | 64.7 |
| Tungsten-containing aldehyde:ferredoxin oxidoreductase (EC 1.2.7.5) | peg1086 | MCF3653595.1 | AOR3_r | GCTGAACTTGCAGAACGTCA | 64.7 |
| Tungsten-containing aldehyde:ferredoxin oxidoreductase (EC 1.2.7.5) | peg1129 | MCF3653121.1 | AOR4_f | GGATTCGCCATGGAACTCTA | 61.4 |
| Tungsten-containing aldehyde:ferredoxin oxidoreductase (EC 1.2.7.5) | peg1129 | MCF3653121.1 | AOR4_r | ATCGAGCCCTTTCCAATTCT | 61.4 |
| Tungsten-containing aldehyde:ferredoxin oxidoreductase (EC 1.2.7.5) | peg1177 | MCF3653435.1 | AOR5_f | GCATGATCCAAGGGCTTTTA | 58.4 |
| Tungsten-containing aldehyde:ferredoxin oxidoreductase (EC 1.2.7.5) | peg1177 | MCF3653435.1 | AOR5_r | ATCCTACCCTTCCCATCCAC | 58.4 |
| Tungsten-containing aldehyde:ferredoxin oxidoreductase (EC 1.2.7.5) | peg1318 | MCF3653347.1 | AOR6_f | GCTCCTCGAGCTTATCATCG | 61.4 |
| Tungsten-containing aldehyde:ferredoxin oxidoreductase (EC 1.2.7.5) | peg1318 | MCF3653347.1 | AOR6_r | TCTGGAGAAGTCCCACATCC | 61.4 |


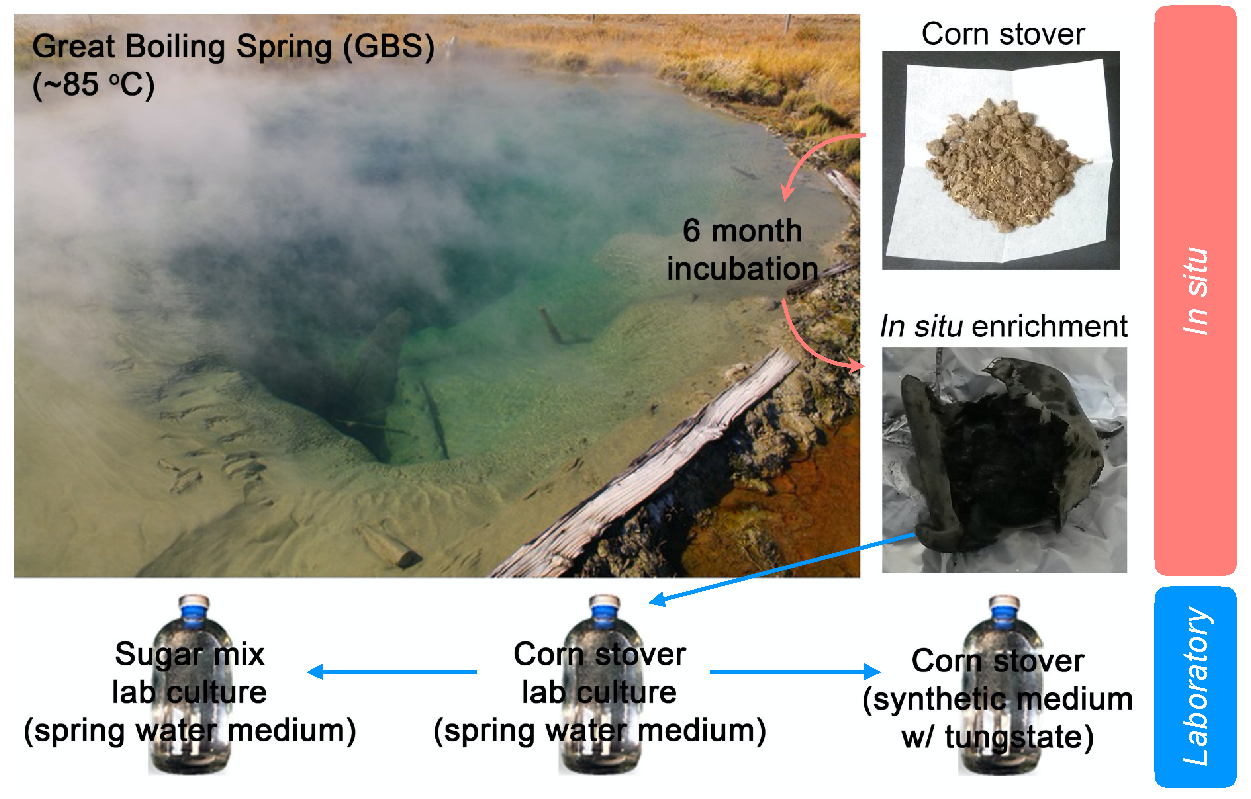


**Supplementary Fig. 1. Cultivation Schematic.** After 6 months of incubation in GBS sediment, corn stover *in-situ* enrichments were inoculated into corn stover medium from which sugar mix lab cultures and synthetic medium lab cultures were derived.


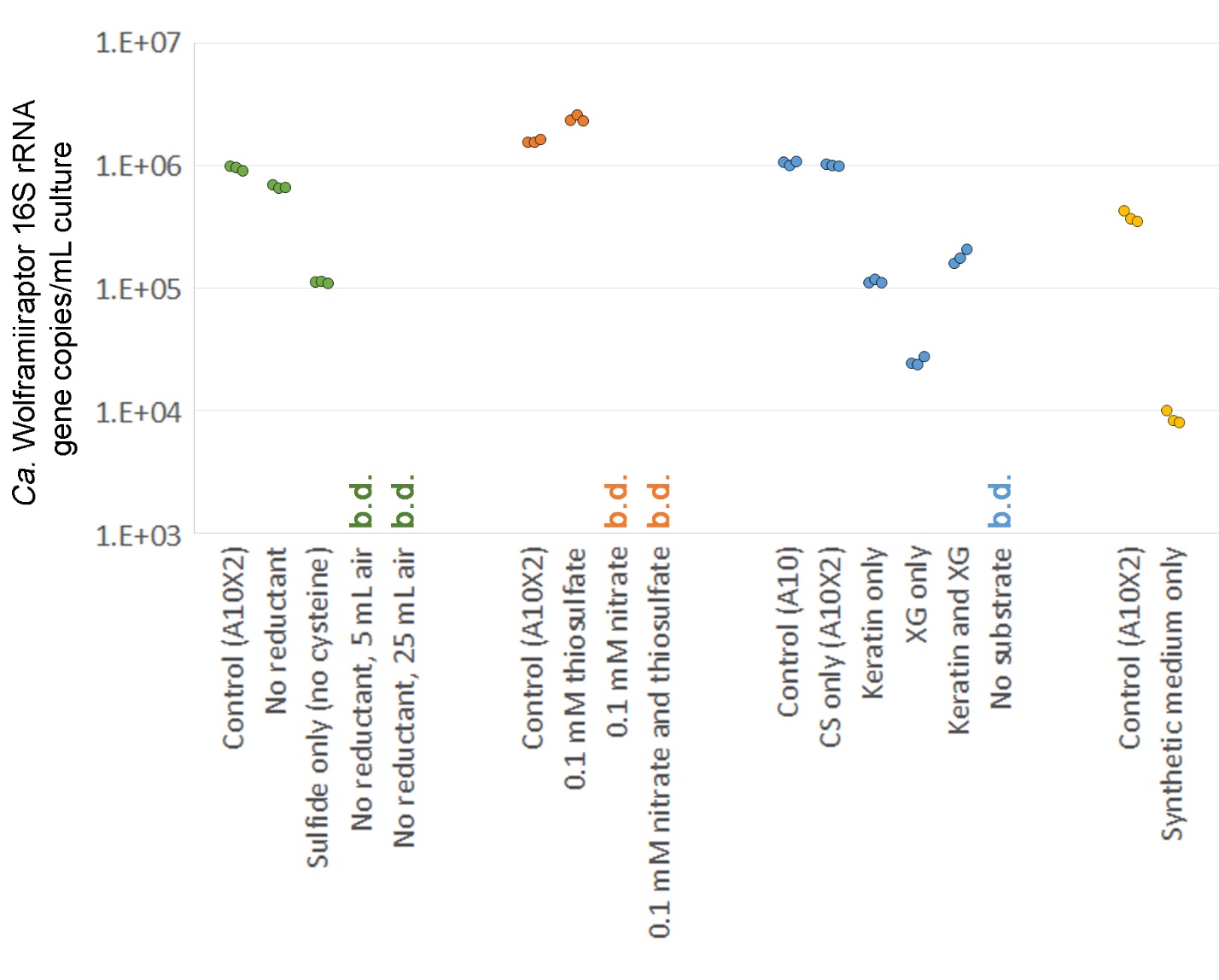


**Supplementary Fig. 2.** **Growth experiment testing for optimal media composition and substrate.** Separate experiments testing growth with different reductants or air (green), effects of thiosulfate and nitrate (orange), growth with individual substrates (blue), and growth in synthetic medium (yellow). AigG4 16S rRNA gene copies/mL culture were quantified by qPCR after two transfers (or one transfer for effects of reductant/air). Quantities below detection of 10^3^ copies/mL are indicated as “b.d.”. Refer to Supplementary Table 1 for control media conditions (A10 and A10x2). Data represent triplicate technical replicates from individual cultures. Corn stover, CS; xyloglucan, XG.


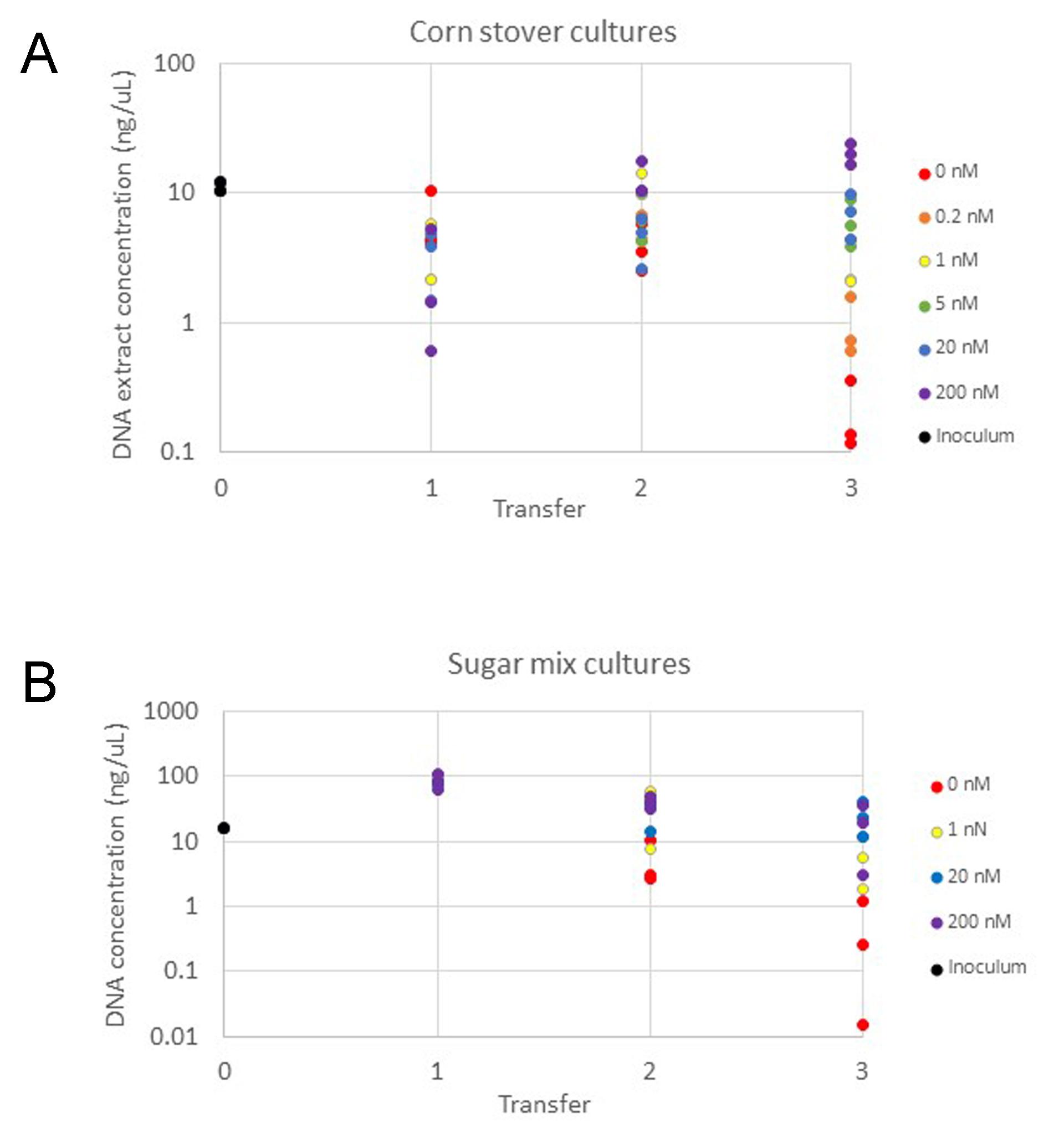


**Supplementary Fig. 3.** **Effects of tungsten on DNA concentrations in extracts from corn stover and sugar mix cultures** shown in Figure 2c (main text). Tungsten concentrations are shown in the legend. DNA concentration in the cultures used for inoculum are also shown.


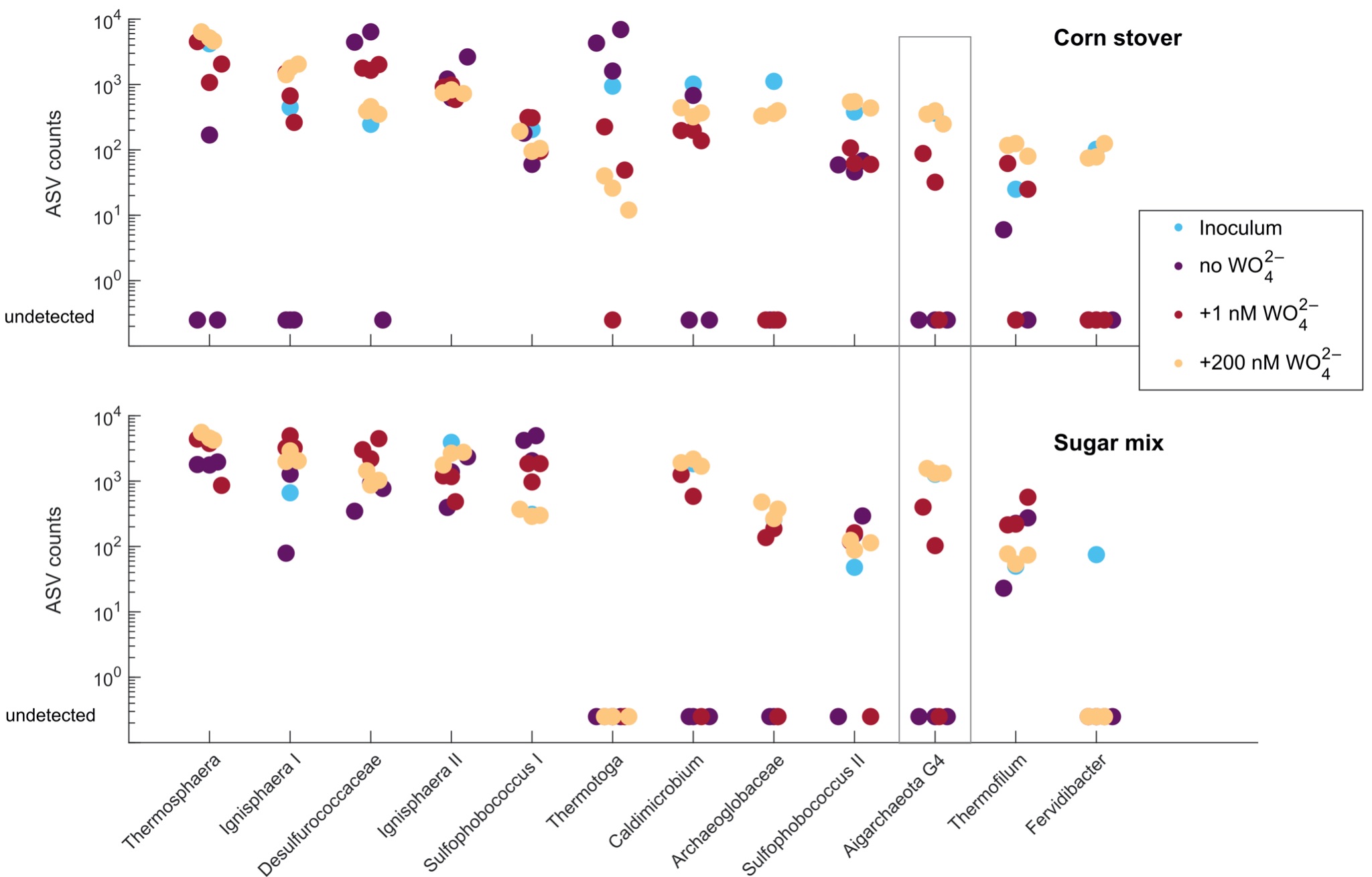


**Supplementary Fig. 4.** **ASV abundances based on 16S rRNA gene tags from synthetic medium amended with either corn stover or a sugar mix under varying tungsten concentrations.** The 12 most abundant taxa are shown, which consistently made up >98% of the total ASVs. *W. gerlachensis* (Aigarchaeota G4) was not detected in any of the replicates without tungsten and showed reduced abundances with low (1 nM) amounts of the trace metal added.


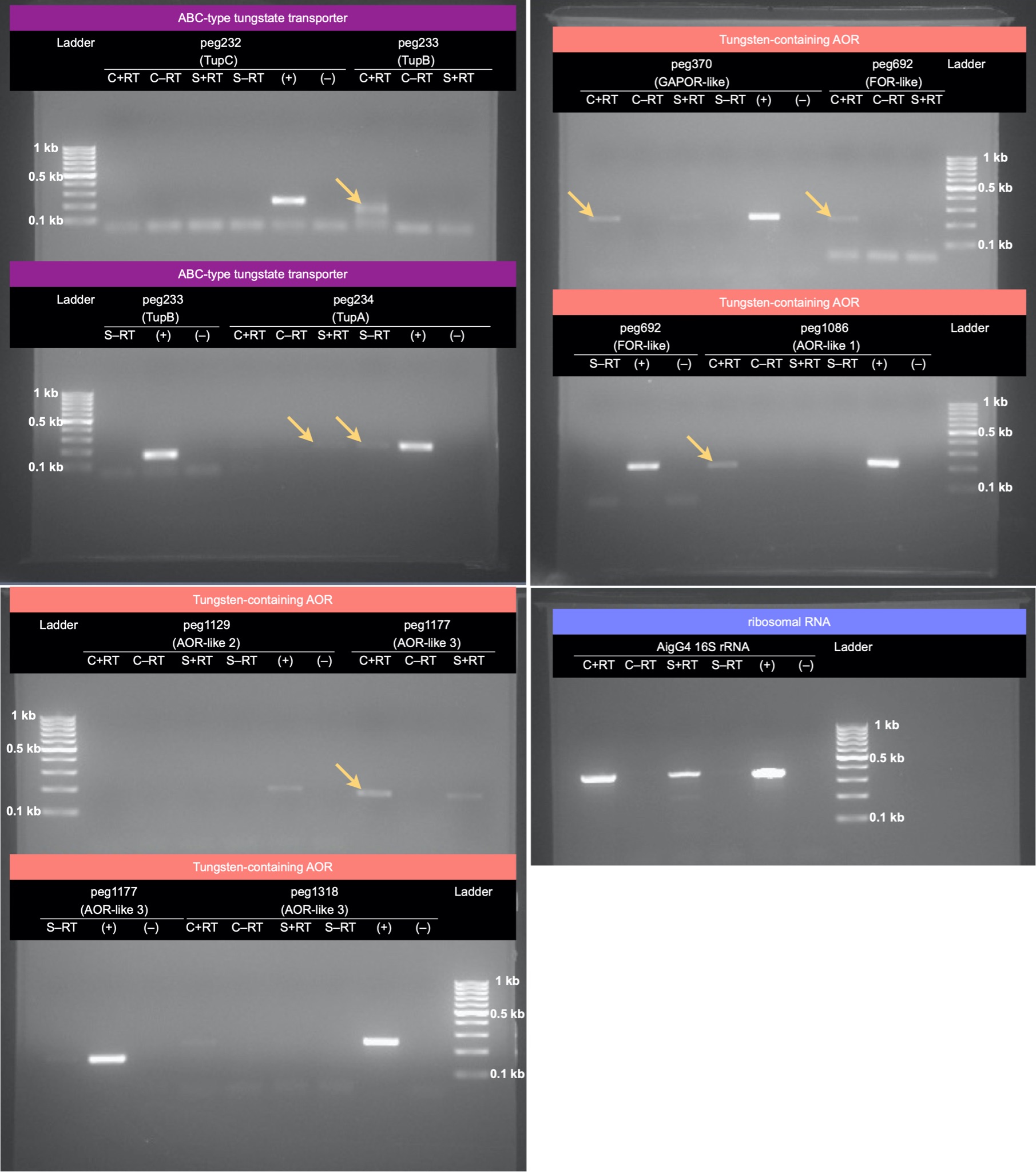


**Supplementary Fig. 5.** **RT-PCR results after 22 days of incubation in corn stover (C) and sugar mix (S) lab culture in spring water medium.** Specific primer sets used are shown above each gel image. Controls included positive controls (+), which verified transcriptase activity based on synthesized templates from RAST, and reactions without reverse transcriptase (-RT) to exclude false positives. Ribosomal RNA specific to AigG4 was present in both corn stover and sugar mix lab cultures. Arrows mark bands that are not positive controls and can be faint. The ladder indicates increments of 100 bp. RAST annotations used here are available under protein accession numbers MCF3653658.1 (peg.232), MCF3653659.1 (peg.233), MCF3653660.1 (peg.234), MCF3653440.1 (peg.370), MCF3653608.1 (peg.692), MCF3653595.1 (peg.1086), MCF3653121.1 (peg.1129), MCF3653435.1 (peg.1177), and MCF3653347.1 (peg.1318). Data was derived from a single experimental run.


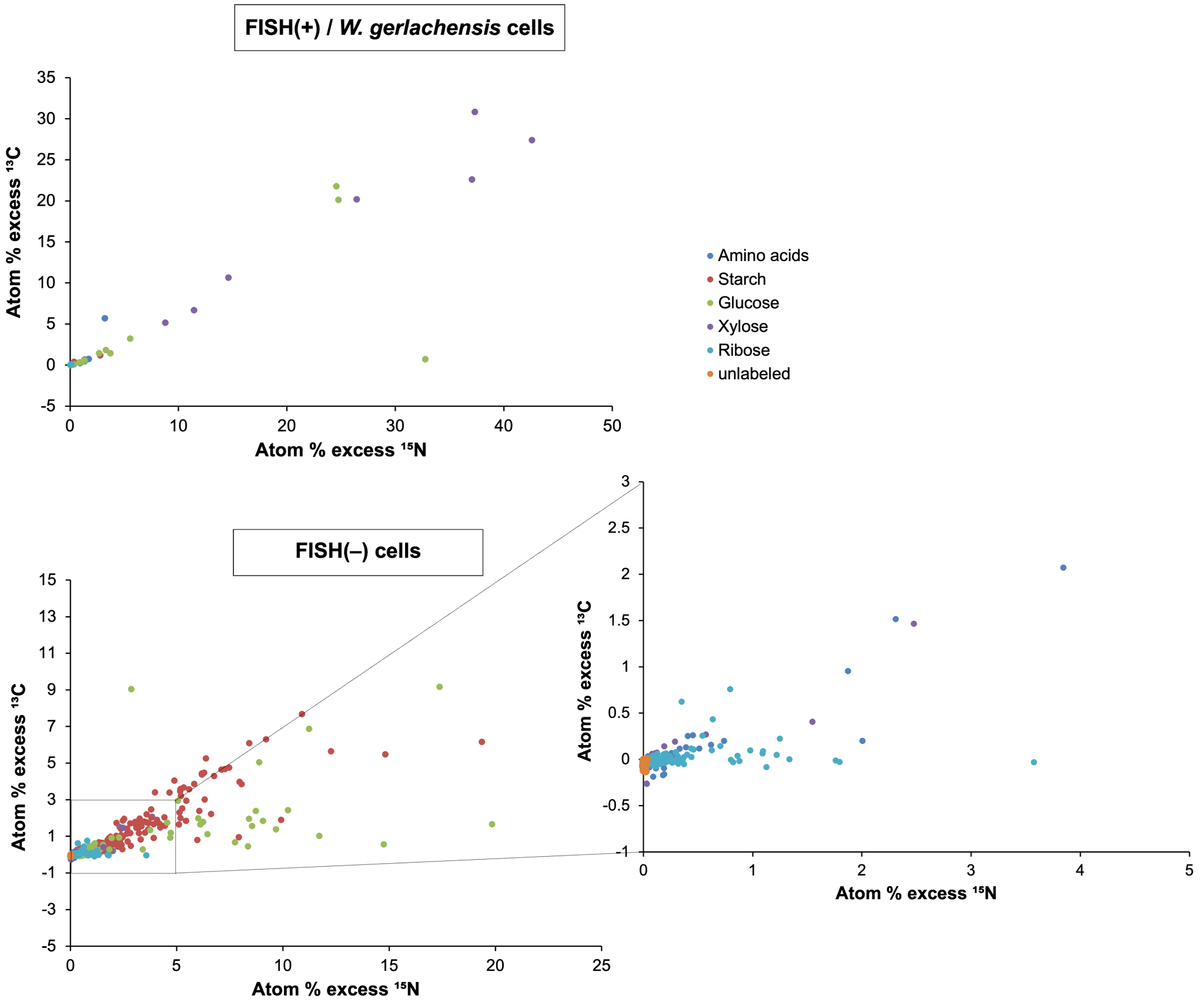


**Supplementary Fig. 6. ^13^C and ^15^N enrichments in FISH(+) and FISH(–) cells.** *W. gerlachensis* cells show correlated assimilation of both isotopes. The FISH(–) cell population also assimilated ^15^N independent of the added carbon substrates, presumably together with unlabeled carbon compounds.

**
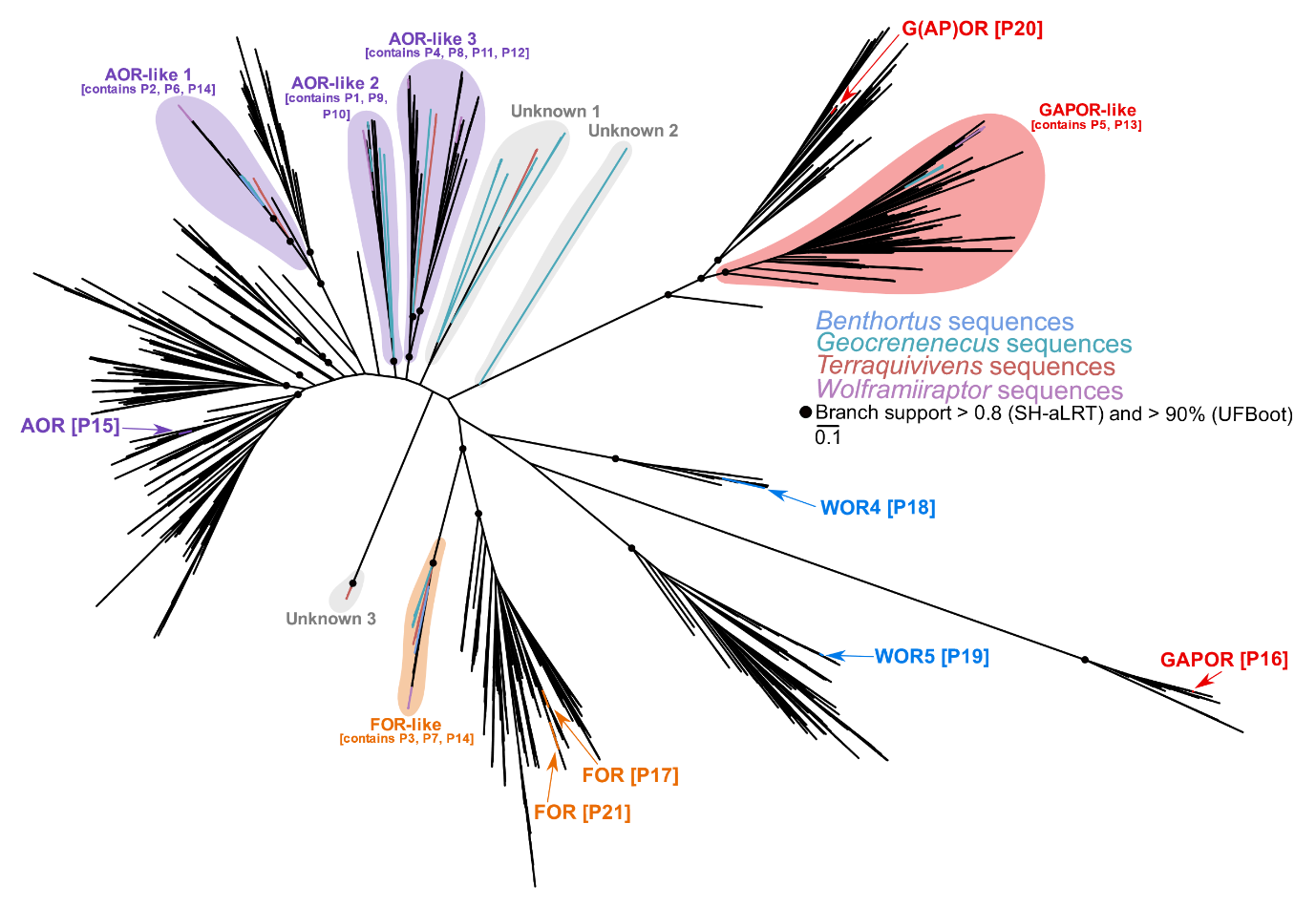
**

**Supplementary Fig. 7.** **Alternate topology of the phylogeny for tungsten-dependent oxidoreductases**. Maximum-likelihood phylogenetic tree of oxidoreductase sequences on the amino acid level retrieved from MAGs belonging to *Wolframiiraptoraceae*, and reference sequences of characterized oxidoreductases. All sequences for *Wolframiiraptoraceae* are indicated in the same colors as Fig. 5 and Fig. S5. Branch-support was inferred with SH-aLRT and Ultrafast bootstrapping and support at deeper nodes among the lineages in the phylogeny are indicated. W-dependent ferredoxin oxidoreductase lineages were inferred based on the topologies of the full unmasked alignment (masked alignment in Figure 4a). The scale bar indicates the number of amino acid changes per site. The full phylogeny is available as Supplementary File S3.


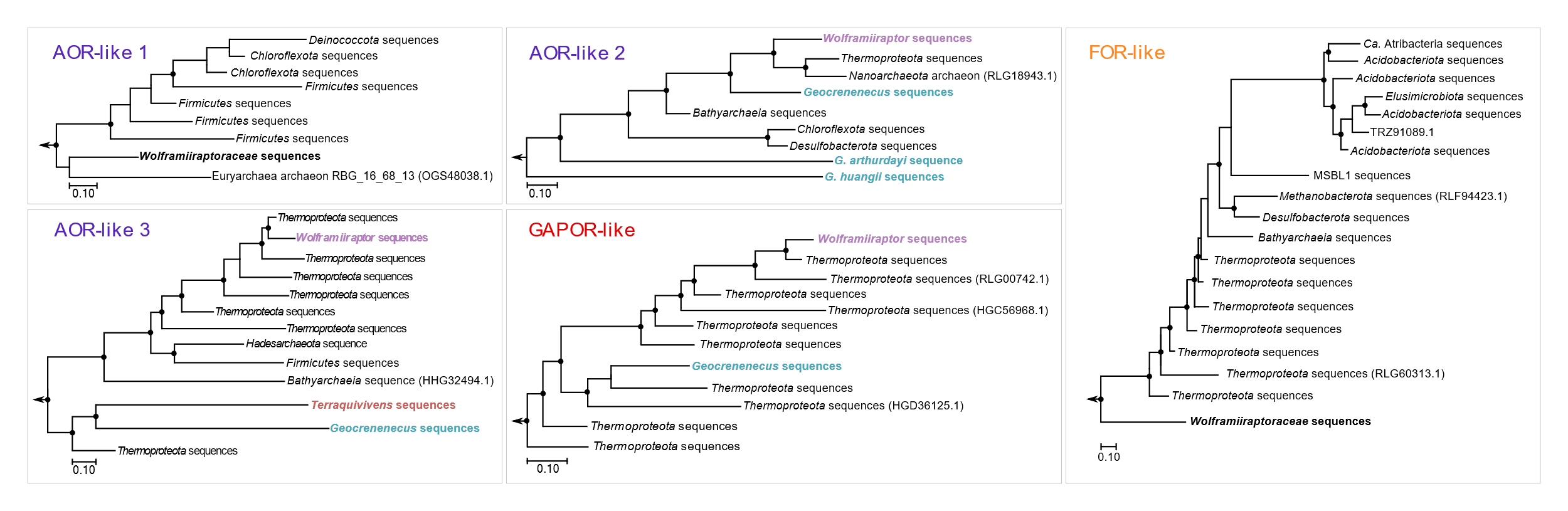


**Supplementary Fig. 8. Summary of tungsten-dependent ferredoxin oxidoreductase lineages.** Summary of subtrees depicting the putative assigned functional lineages based on phylogeny and structural modeling analysis. Multiple sequences belonging to the same phylum are compressed and represented as a single terminal taxon. Supported branches (> 0.8 SH-aLRT and > 90 % Ultrafast bootstrap) are indicated with filled circles at nodes. Sequences derived from members of the *Wolframiiraptoraceae* are indicated in bold, while genera are colored according to their respective colors where the sequences from the family were not recovered as a monophyletic group. For full phylogeny in newick format, see supplementary File S2.


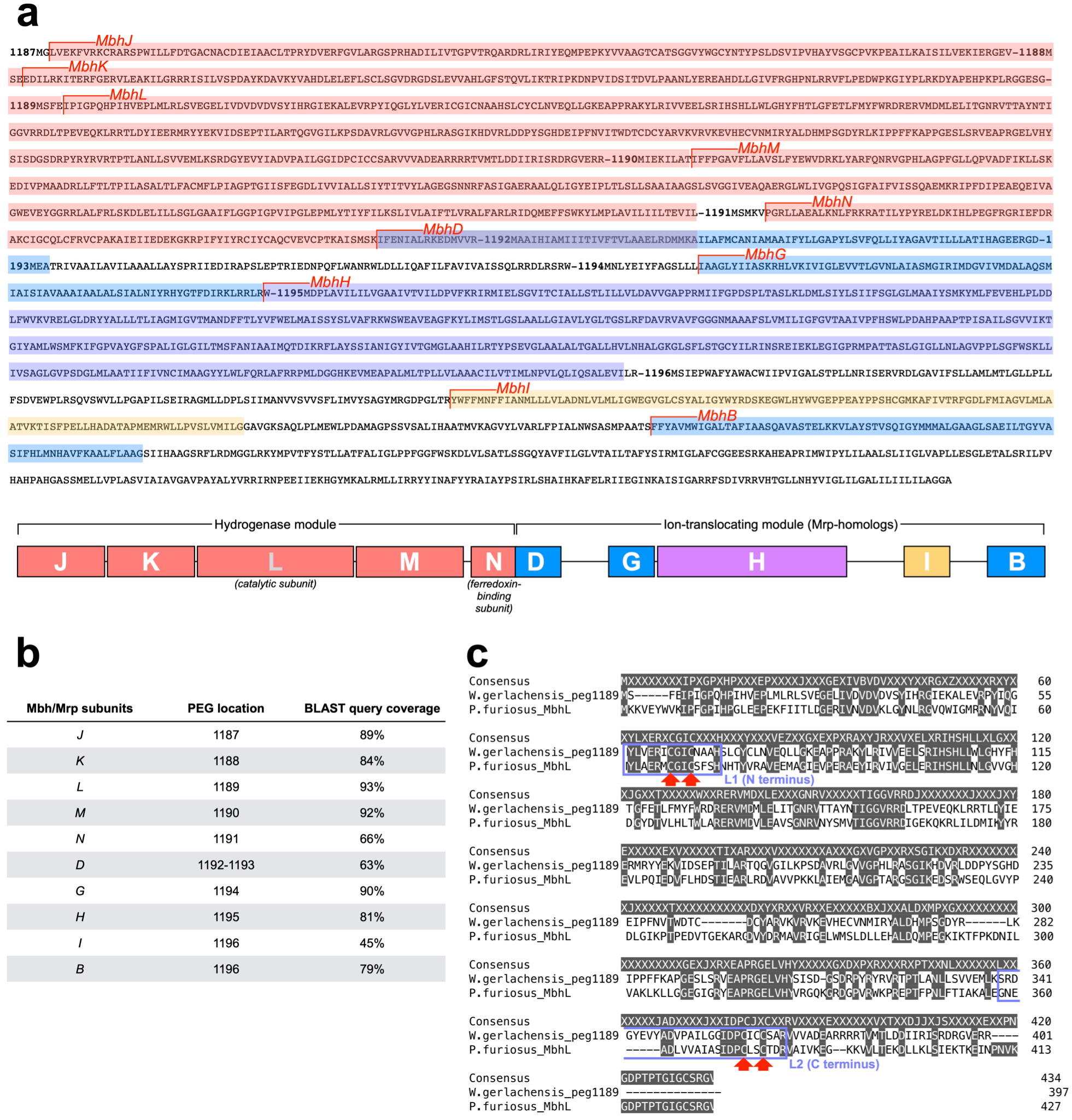


**Supplementary Fig. 9.** **Sequence homologs of the Mbh complex are present in the *W. gerlachensis* genome. a,** Co-located genes of 10 Mbh subunits were identified including all subunits of the hydrogenase module. **b,** BLAST alignments were conducted using *Pyrococcus furiosus* queries of the respective Mbh/Mrp genes. Coverage was generally >60% (except for one subunit). **c,** The conserved cysteine residues within the N and C terminus of the catalytic subunit MbhL (*P. furious*) are present on MCF3653637.1 (peg.1189) (*W. gerlachensis*).


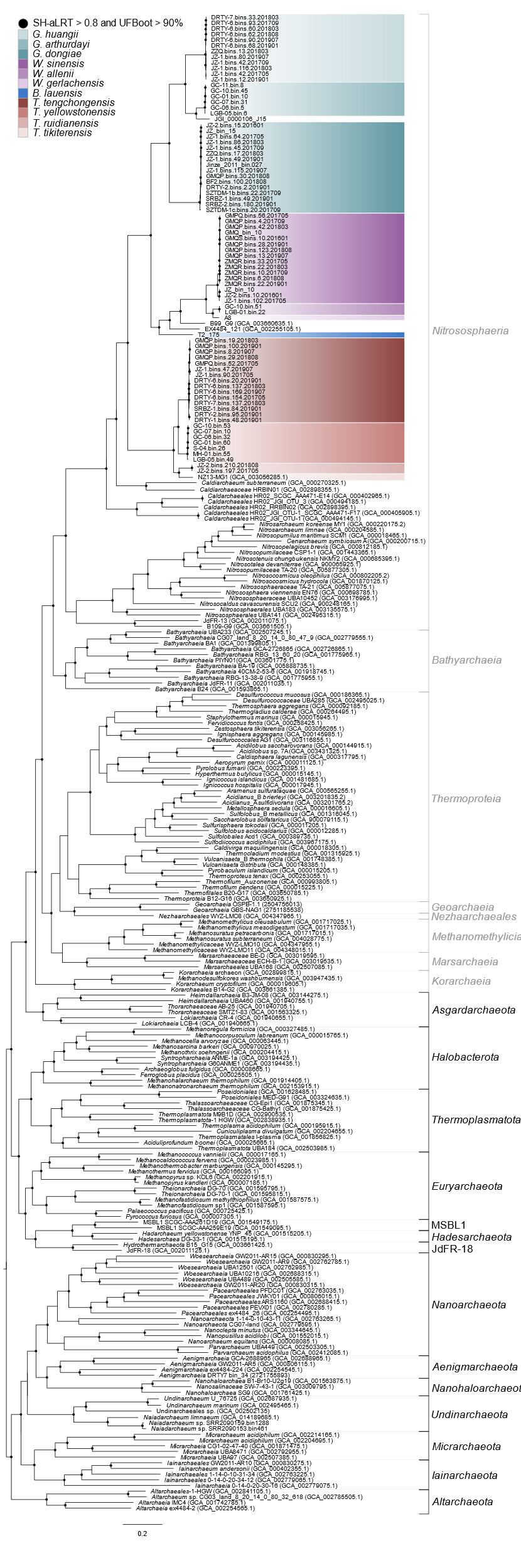


**Supplementary Fig. 10.** **Archaeal phylogeny placing the family *Wolframiiraptoraceae* in a phylogenetic context.** The taxon set was constructed using the representative genome set of Dombrowski et al., 2020, and supplementing this taxon set with representative genomes within the *Thermoproteota* and publicly available genomes for the order *Caldarchaeales*. The phylogeny was based on 122 conserved archaeal proteins and was inferred from a partitioned, concatenated matrix using IQ-Tree and branch-support was inferred with SH-aLRT and Ultrafast bootstrapping. Archaeal phyla are annotated on the phylogeny in black, while grey annotations indicate putative class-level lineages within the *Thermoproteota*.


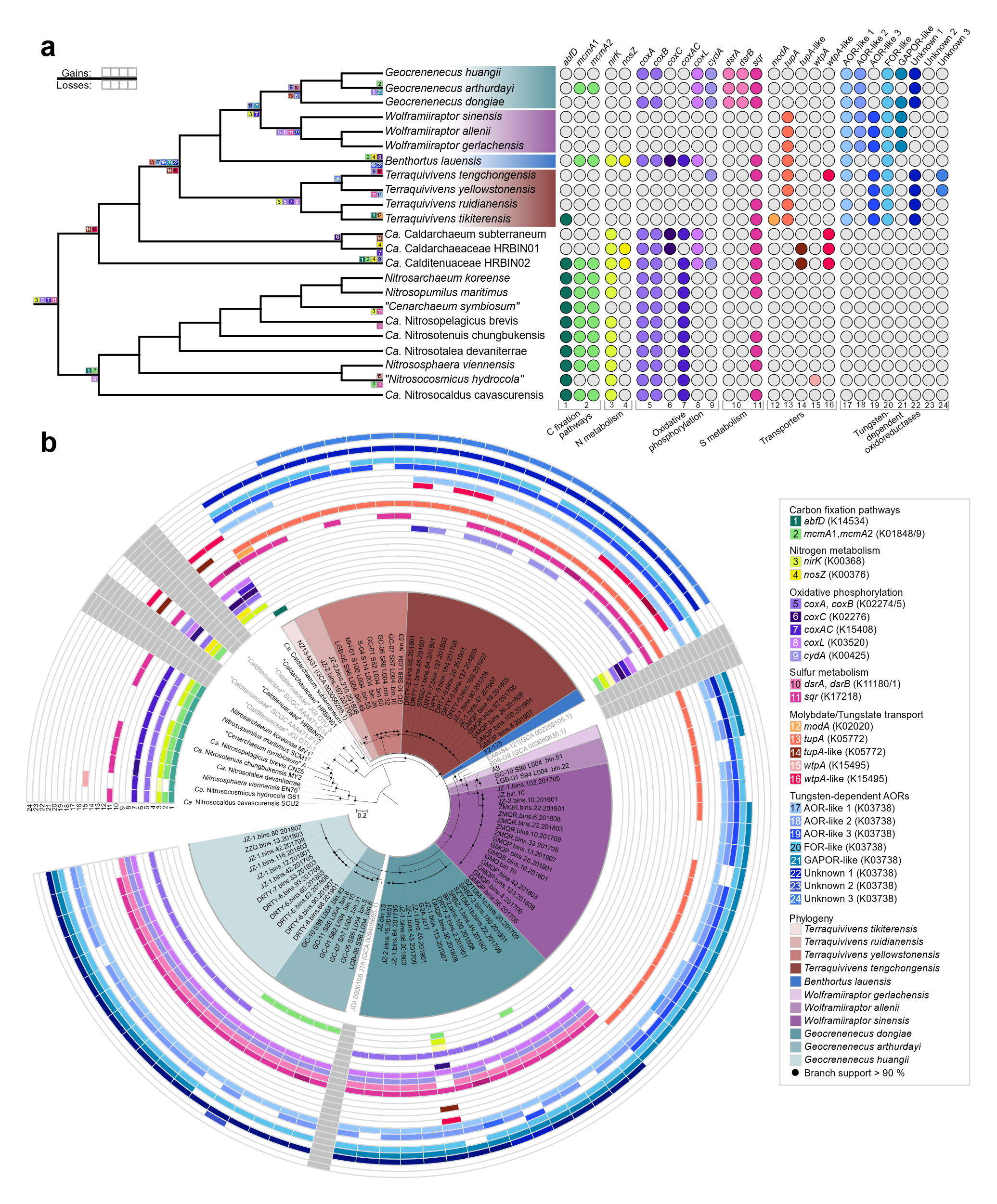


**Supplementary Fig. 11.** **Ancestral state reconstruction (a) and presence/absence of key genes in all genomes of the family *Wolframiiraptoraceae* (b). a,** Cladogram inferred from the Maximum likelihood phylogeny for the family, with predicted gene gains or losses of genes involved in carbon fixation pathways, nitrogen metabolism, oxidative phosphorylation, sulfur metabolism, tungstate and molybdate transporters and tungsten-dependent oxidoreductases. Lighter shades in the dot plot indicate presence of genes in one or some members of the species, but lacking in the majority of the members of the species. For the COUNT session file of ancestral reconstruction, please see Supplementary File S2. **b,** Distribution of genes related to physiology, including tungsten-associated genes within the genomes of *Wolframiiraptoraceae*. Darker shades indicate presence in multiple copies of genes in the specific genomes. Medium-quality genomes are indicated in grey in the phylogeny, and were not used in presence or absence analyses.

**
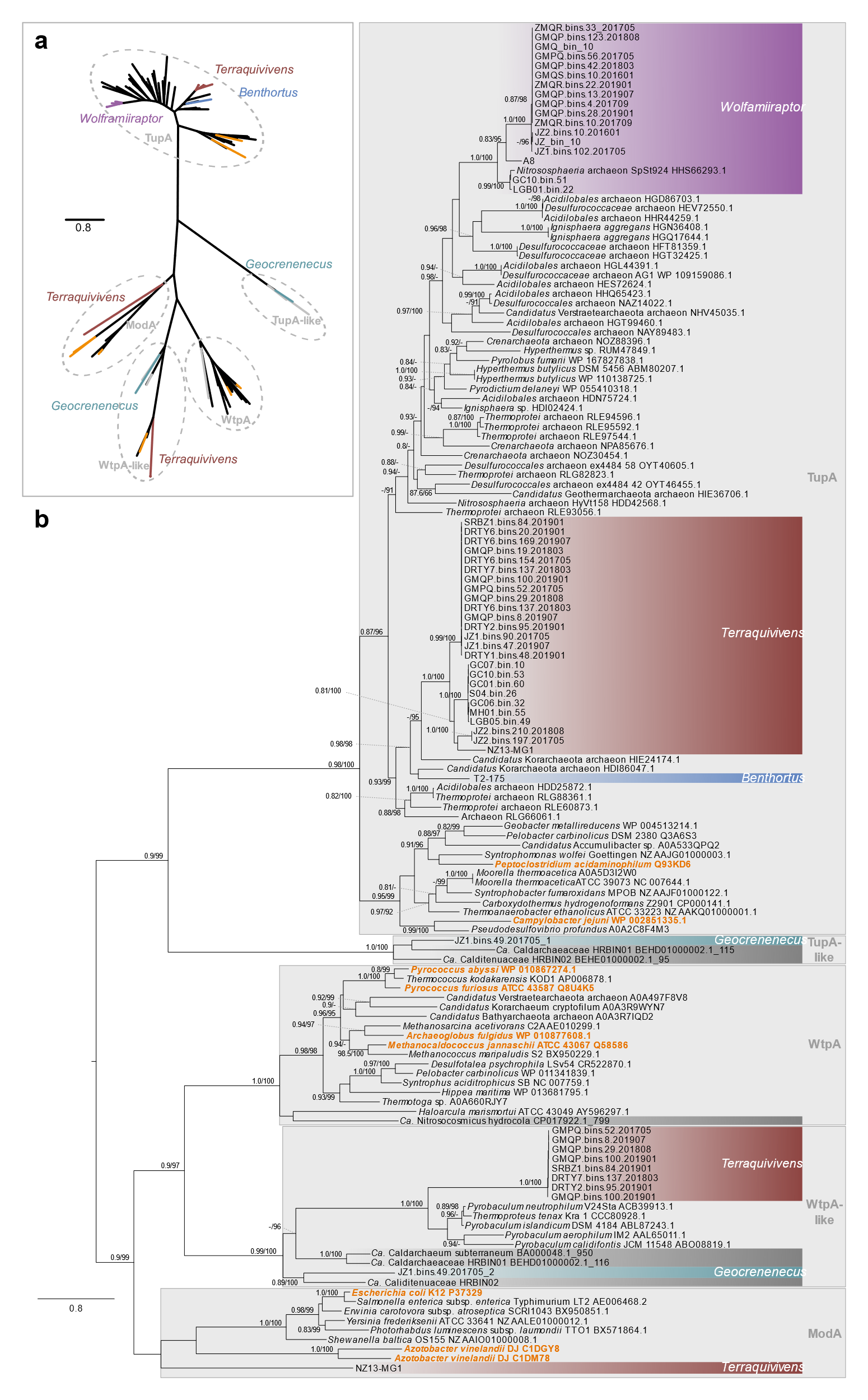
**

**Supplementary Fig. 12.** **Phylogeny of all substrate-binding subunits of molybdate and tungstate ABC transporters in *Wolframiiraptoraceae*.** These sequences include all sequences annotated as encoding subunit A of the Tup (tungstate uptake ABC transporter; K05772), Wtp (tungstate/molybdate ABC transporter; K15495), and Mod (molybdate ABC transporter; K02020) systems. Alignment of sequences were done with DASH as implemented in MAFFT, and a maximum-likelihood phylogeny was constructed with IQTree. Phylogenetic placement of sequences derived from genomes of members of *Wolframiiraptoraceae* are indicated in colors corresponding to the genera. Orange branches and taxon names indicate reference sequences for the respective ABC transporter systems with functional evidence available, while grey sequences represent those from outgroup taxa (other genomes of *Caldarchaeales* and the *Nitrososphaerales*). The reference sequences included *Campylobacter jejuni* and *Peptoclostridium acidaminophilum* for the TupA homologs; *Archaeoglobus fulgidus*, *Methanocaldococcus jannaschii*, *Pyrococcus abyssi* and *Pyrococcus furiosus* for the WtpA homologs; *Pyrobaculum islandicum*, *Pyrobaculum aerophilum* and *Pyrobaculum caldifontis* for the WtpA/ModA-like sequences; and *Escherichia coli* and *Azotobacter vinelandii* for the ModA homologs. The scale bar indicates the number of amino acid changes per site. **a)** Summarized unrooted tree for the A subunits of transporters identified from the genomes. **b)** Support values derived from 1,000 replicates with SH-aLRT and UFbootstrapping are indicated at nodes if SH-aLRT were above 0.8, and UFbootstrap was above 90 %.


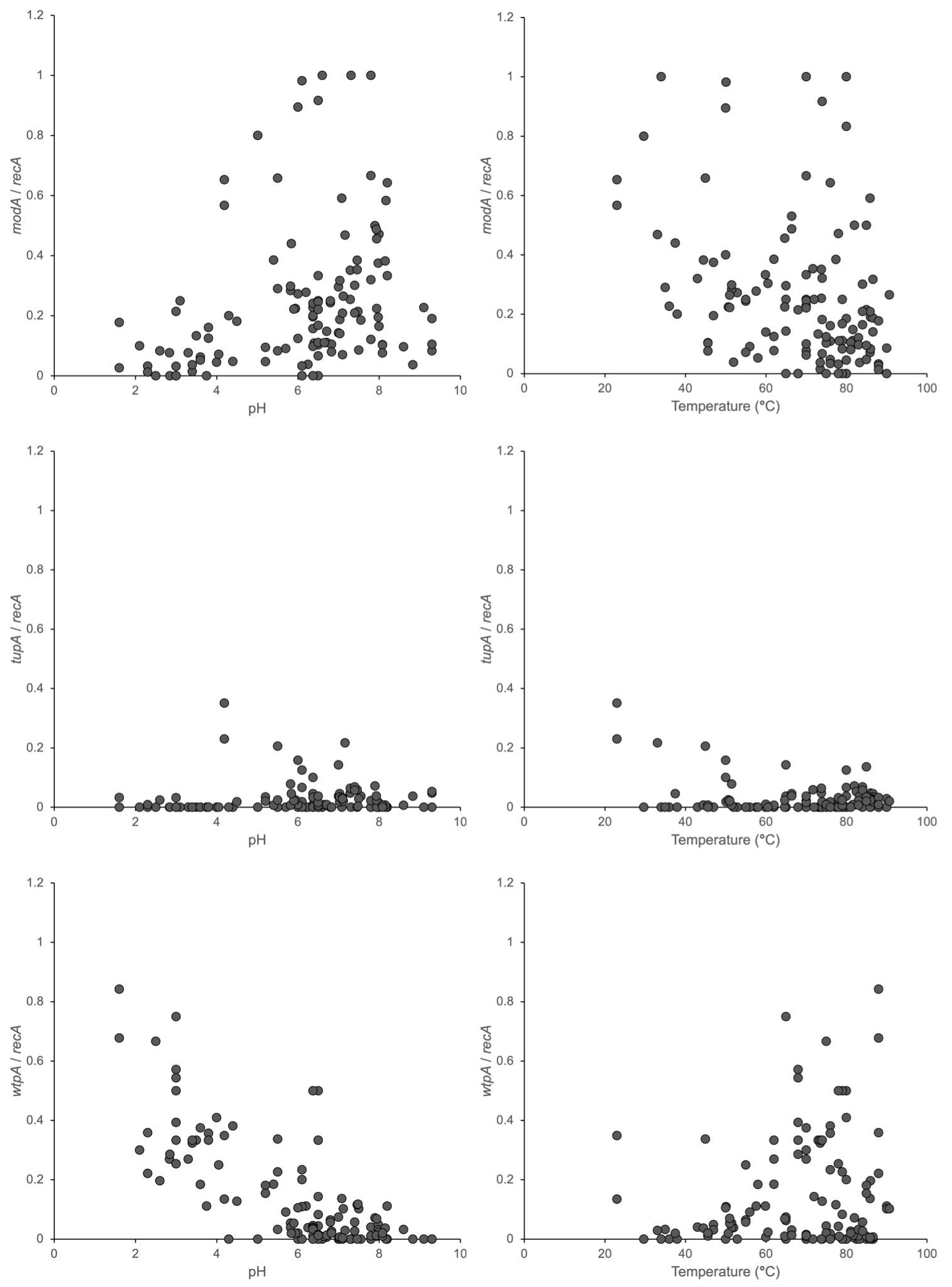


**Supplementary Fig. 13. Relative *modA*, *tupA*, and *wtpA* gene frequencies in metagenomes derived from hot springs with different pH and temperature conditions.** Gene counts were normalized to the housekeeping gene *recA* to enable comparison between the transporter types. Only contemporaneous temperature and pH data was used. The tungsten transporters show particular presence in waters above 60 °C. *WtpA* is generally more abundant than *tupA* and is most abundant in low pH (< 4) hot springs.

**
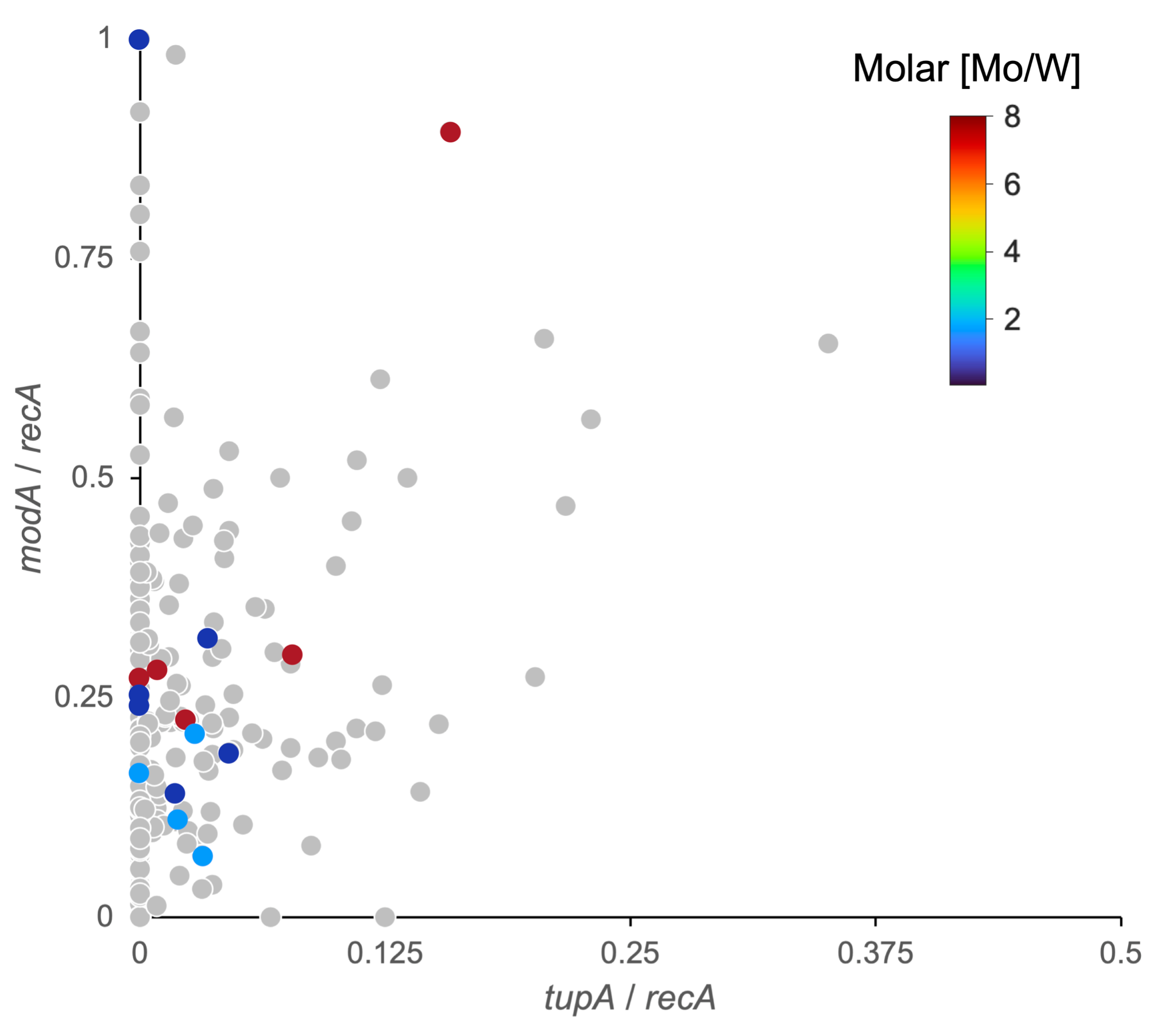
**

**Supplementary Fig. 14. Relative *modA* and *tupA* gene frequencies with marked molar concentration ratios of Mo and W in the spring water.** Due to a lack of contemporaneous trace metal data, we were not able to infer a correlation of trace metal abundances with transporter gene frequencies.

***Supplementary Note 1: Additional information on enrichment, sampling, and metagenomic DNA extraction***

For *in situ* enrichments in Great Boiling Spring, Nevada, USA, approximately 0.5 g of corn stover was sealed in 100 µm nylon mesh bags, placed in 50 mL plastic conical tubes with holes drilled in them for exchange with spring water, and placed at the sediment/water interface at the "A site"14 of GBS on 15 September 2015. Temperature and pH of spring water immediately above the incubation site were measured using a LaMotte pH5 Series pH/temperature meter (LaMotte, Chestertown, MD, USA). *In situ* corn stover enrichment that remained after sampling for cultivation experiments was placed in 1.5 mL centrifuge tubes, frozen immediately and transported back to the laboratory on dry ice, and then stored at -80 °C for DNA extraction. In lab cultures, further experiments indicated that *W. gerlachensis* growth could, other than by corn stover, also be supported by several different individual sugars or a mix of sugars (0.01% each of glucose, xylose, mannose, and D- and L-arabinose; “sugar mix”) with approximately 10-fold higher abundance (~10^7^-10^8^ 16S rRNA gene copies mL^–1^ culture), but at a similar relative abundance. The lab cultures were used for multiple attempts to obtain axenic cultures (e.g., using gelrite plates, dilution cultures in liquid medium); however, no pure cultures or highly enriched mixed cultures were obtained.

A deep-sea hydrothermal sulfide deposit (J2-819-10-R1) was collected with the ROV Jason from the Tu’i Malila vent field along the Eastern Lau Spreading Center-Valu Fa Ridge (21°59.42496’S, 176° 34.15134’W, 1840 m depth) during the April/May 2015 RR1507 expedition aboard the RV Roger Revelle. The sample was collected and prepared as described previously^1^ and stored at -80 °C before DNA extraction.

All samples taken from hot springs in Tengchong, Yunnan, China, were derived from sediment samples. Shortly, sediments were sampled with sterile spatulas and spoons. Samples were frozen and stored in liquid nitrogen, followed by storage at -80 °C before DNA extraction. Temperature and pH measurements were taken at the time of sampling (see File S1 for details for MAGs).

For all samples taken from hot springs in Yellowstone National Park, USA, sediment samples were sterilely collected for metagenomic analyses in 2019 and 2020. Samples were immediately placed on dry ice for transport to the laboratory, followed by subsequent storage at -80 °C.

DNA extraction protocols differed for the different sediment or deposit samples used for metagenomic sequencing. In addition to those protocols described in the main text, the following methods were used. For the marine hydrothermal sulfide deposit sample, the Qiagen DNeasy PowerSoil kit was used to extract DNA, using manufacturer’s recommendations. Approximately 20 g of sediment from each sample from the geothermal springs in Tengchong, China, was used for genomic DNA extraction. All extractions from sediment samples from China were performed with the Powersoil DNA Isolation Kit (MoBio), except where FastDNA SPIN Kit (MP Biomedical, Solon, OH, USA) were used for the extraction DNA for the sample for G2A_Jz17, following the manufacturer’s recommendations, and DNA concentrations were measured using a Qubit fluorometer. For samples taken from Yellowstone National Park, USA, DNA extractions were conducted as previously described^2^. Briefly, at least duplicate extractions were conducted from ~0.5 g of sediment using the FAST DNA Spin Kit for Soil (MP Biomedicals, Irvine, CA) following the manufacturer’s protocols. Extracts were then pooled as needed to achieve enough DNA for shotgun metagenomics sequencing.

***Supplementary Note 2: Metagenomic sequencing, assembly, and binning of metagenome-assembled genomes***

Seventy-eight MAGs were identified as belonging to the family *Wolframiiraptoraceae*, from various thermal aquatic environments, and encompass four candidate genera containing eleven species. A total of 60 high-quality MAGs representing five putative species, were identified from geothermal springs from the Rehai and Ruidian geothermal fields, Tengchong, China, while 15 high-quality MAGs representing three putative species were identified from Yellowstone National Park, USA, with all eight putative species belonging to three genera. A single high-quality MAG, representing a putative species, was obtained from Tikitere, Rotorua, New Zealand, while another MAG, representing a putative species in a novel genus, was obtained from a hydrothermal vent from the Lau Basin, in the Pacific Ocean. One high-quality hybrid MAG assembly was obtained from long- and short-read sequencing runs of a stable enrichment culture (see below) established from Great Boiling Spring, NV, USA. Overall, the high-quality genomes analyzed in this study ranged in quality estimates from 90.29 to 99.03 %, with 0 to 3.47 % contamination. All MAGs were classified as belonging to the family f__NZ13-MGT, in the order *Caldarchaeales* using GTDB-Tk.

Shotgun metagenome sequencing using the Illumina MiSeq platform (2x250 bp) was done on a subculture of the A8X3 culture at Argonne National Laboratories, with library preparation using the Nextera Flex DNA kit. For Oxford Nanopore sequencing, libraries were prepared on DNA extracted from “sugar mix” cultures containing “AigG4” following the Native Barcoding (EXP-NBD103) and Ligation Sequencing (SQK-LSK108) Kit 1D protocols according to manufacturer’s instructions, and sequencing was performed on a MinION FLO-MIN106 flow cell (Oxford Nanopore, Oxford Science Park, UK). Long Nanopore sequencing reads were assembled with metaFLYE v. 2.8.2^3^, using the --nano-raw and --meta options. The obtained long-read assembly was then used as untrusted contigs for assembly of Illumina reads using SPAdes v. 3.14.1^4^. The obtained hybrid assembly was assessed with metaQUAST v. 5.0.2^5^. This assembly was then subjected to binning with MetaBAT2 v. 1.7^6^ and MaxBin2 v. 2.2.4^7^, as implemented in KBase^8^. To assess the binning efficiency of both binning algorithms used for the GBS-derived hybrid assembly, contig coverage, as determined based on the Illumina sequence data, was plotted against contig length within the binned MAGs. This was followed by assessment of MAG completeness and contamination using CheckM^9^, and classification using the GTDB Toolkit v. 1.1.0^10^, as implemented in KBase^8^. MAGs identified as belonging to the order *Caldarchaeales* through GTDB-Tk were used to plot contig coverage against contig length, to compare the binned genomes to one another (data not shown). Initial annotation of the combined *Caldarchaeales* hybrid assembly MAG was performed using RAST^11^.

For the hydrothermal sulfide deposit sample, a metagenomic library was prepared with an Illumina Nextera library prep kit and sequenced (2 x 150 bp) on the Illumina HiSeq3000 at the Center for Genome Research and Biocomputing, Oregon State University. Low-quality reads and adaptor sequences were removed from the metagenome using Trimmomatic v.0.36^12^, and the khmer software package was used to interleave reads. An assembly was then performed using MEGAHIT v.1.1.1-2-g02102e1^13^ (--k-min 31, --k-max 151, --k- step 20, --min-contig-len 1000), and a coverage profile was generated by mapping reads back to the contigs with bowtie2 v.2.2.9^14^ and SAMtools v.1.3.1^15^. Both coverage and tetranucleotide frequency were used to bin the assembly with MetaBAT v.0.32.4^16^ (only contigs ≥1500 bp retained), and CheckM v.1.0.7^9^ was used to assess completeness and contamination.

Approximately 30 Gbp (2 × 150 bp) of metagenomic data for each sediment sample taken from Chinese hot springs, was generated by using the Illumina HiSeq 4000 Platform using a 350 bp insert library at Beijing Novogene Bioinformatics Technology Co., Ltd (Beijing, China). The raw reads were quality filtered as described earlier^17^, by eliminating adapter-contaminated reads, deleting PCR-generated duplicated reads, removing reads with a significant excess of “N” (≥ 10% of the read), and trimming reads with a quality score of less than 15 at the 3′ end. The high-quality reads of each sample were de novo assembled individually, using metaSPAdes v. 3.9.0^4^ with the following kmers: -k 33, 55, 77, 99, 111. Reads were mapped to scaffolds using BBMap v. 38.85^18^. Genome binning was conducted on scaffolds with length > 2.5 kbp using MetaBAT^16^. The estimated quality of binned MAGs were evaluated using CheckM^9^, and classification was done using the GTDB Toolkit v. 1.1.0^10^ to identify MAGs belonging to *Caldarchaeales*.

Yellowstone hot spring metagenomes were produced on the Illumina NovaSeq platform 2x150 bp). Sequencing adapters and quality filtering of raw reads was conducted using the TrimGalore v.0.6.0^19^ implementation of the Cutadapt pipeline v.2.1.0^20^ with default parameters. Quality-filtered reads from both datasets were first down-sampled to minimize data redundancy and mitigate uneven population sequencing depth coverage using the BBnorm script (https://github.com/BioInfoTools/BBMap)^18^, as described elsewhere^21^. Down-sampled reads were then assembled using the MetaSPAdes v.3.14.0^4^ with a range of k-mer lengths and default parameters. Assembled contigs were then subjected to metagenome-assembled-genome (MAG) binning using the MetaWRAP v.1.3.2 pipeline^22^. Briefly, read mapping to the assembled contigs was conducted with the BWA aligner v.0.7.17^23^, followed by binning of contigs > 2.5 kbp in length with the MetaBAT2^6^, MaxBin2^7^, and CONCOCT v.1.1.0^24^ algorithms. The “bin_refinement” module of MetaWRAP was used to identify the highest quality bins among the three binning strategies based on CheckM v.1.1.3^9^ estimates of completeness and contamination. Only those MAGs exhibiting > 90 % estimated completeness and < 5 % estimated contamination are reported here.

***Supplementary Note 3: Archaeal phylogeny***

A taxon set representative of the domain Archaea was constructed using the 127 representative archaeal genomes as identified in Dombrowski et al.^25^, and supplementing this taxon set with a further 59 genomes of type strains for genera within the phylum *Thermoproteota*. Furthermore, all publicly available genomes identified as belonging to the *Caldarchaeales* through the GTDB release 95, along with the 78 genomes designated here as members of the *Wolframiiraptoraceae* were included for phylogenetic analysis. Using the GTDB Toolkit v. 1.4.1^10^, 122 archaeal conserved markers were identified from the genomes and aligned, followed by inference of the appropriate amino acid model of evolution for each marker with ProtTest v. 3.4^26^, and concatenated and partitioned with FASconCAT-G v. 1.0.4^27^. This concatenated partitioned data matrix was subjected to maximum-likelihood analysis in IQ-Tree v. 1.3.11.1^28^, using the edge-unlinked model with the appropriate model specified for each partition, and branch support inferred using the ultrafast bootstrap analysis^29^ and Shimodaira-Hasegawa-like approximate Likelihood Ratio Test (SH-aLRT; ref. ^30^) from 1,000 replicates each.

For the analysis of all tungstate and molybdate transporters within the family, all sequences annotated as ModA (K02020), TupA (K05772) or WtpA (K15495) with eggNOG were used to construct the transporter dataset. Of these sequences, the TupA sequence encoded by the genome of *W. gerlachensis* A8 was compared against the non-redundant protein (nr) database using BLASTP^31^ with default search parameters. Any hits with ≥50% identity across ≥70% query coverage was considered to represent homologs of the tungsten or molybdate ABC transporter systems Also, all homologs identified in Bevers et al.^32^ as well as other predicted homologs of these systems in literature were added to the datasets, which included the respective gene sequences encoding the tungsten/molybdate (WtpA) and molybdate (ModA) ABC transporter systems. For reference purposes, all functionally verified homologs of the A subunits of the respective ABC transporter systems were also included in the dataset. Alignment for this dataset was conducted with DASH^33^, as implemented in MAFFT^34^.

In order to investigate the phylogenetic placement of predicted tungsten-dependent oxidoreductases present in *Wolframiiraptoraceae*, the protein sequences of all these genes annotated as encoding aldehyde:ferredoxin oxidoreductases (K03738) were extracted and a dataset based on Scott et al.^35^ was constructed. The predicted sequences for *Wolframiiraptoraceae*, along with experimentally verified reference sequences for aldehyde ferredoxin oxidoreductase [AOR (WP_011011461.1)], formaldehyde ferredoxin oxidoreductase [FOR (O93738)], glyceraldehyde ferredoxin oxidoreductase [GAPOR (WP_011011581.1)] and tungsten oxidoreductases of unknown function [WOR4 (WP_011013103.1) and WOR5 (WP_014835423.1)] from *Pyrococcus furiosus*, glyceraldehyde-3-phosphate ferredoxin oxidoreductase [G(AP)OR (B9MQI2)] from *Caldicellulosiruptor bescii* and formaldehyde ferredoxin oxidoreductase [FOR (WP_004066104.1)] from *Thermococcus litoralis*, were used to search the non-redundant database with BLASTP. Default BLAST parameters were employed and all hits with ≥50% identity across ≥70% query coverage were considered homologs of the protein used as query sequence. These blast hits were combined with the *Wolframiiraptoraceae* sequences and the reference sequences into a single dataset. All duplicate sequences, detected via blasts to multiple references or *Wolframiiraptoraceae* sequences were removed. This dataset was then subjected to multiple sequence alignment using the online service of MAFFT v. 7^34^ using the progressive method FFT-NS-1, due to the large size of this dataset (>2,200 sequences).

Key genes relating to the physiology of these microorganisms were identified based on KEGG orthology annotations. These included *abfD* (K14534), *mcmA1* (K01848) and *mcmA2* (K01849), involved in carbon fixation pathways; *nirK* (K00368) and *nosZ* (K00376) involved in nitrogen metabolism; *coxA* (K02274), *coxB* (K02275), *coxC* (K02276), *coxAC* (K15408), *coxL* (K03520), and *cydA* (K00425) involved in oxidative phosphorylation; *dsrA* (K11180), *dsrB* (K11181) and *sqr* (K17218) involved in sulfur metabolism. As reference sequences, KO identifiers of proteins were downloaded from the UniProtKB database database (UniProt Consortium, 2018) and CD-hit v.4.8.1^36^ was applied to dereplicate predicted protein sequences at a 90 %, 80 % (*coxA*), 70 % (*coxC* and *cydA*), or 60 % (*coxB*) similarity. Additionally, closest relatives of these sequences were identified with BLASTP^31^ in the nr database, as described for the tungsten-associated sequences. Multiple sequence alignment was performed as described above.

Maximum-likelihood analyses for all single-gene trees were conducted using IQ-Tree v. 1.3.11.175 or v. 1.6.8^28^, where the appropriate substitution model for each analysis was determined and branch-support was inferred with ultrafast bootstrap analysis^29^ and/or Shimodaira-Hasegawa-like approximate Likelihood Ratio Test (SH-aLRT; ref. ^30^). The phylogenies were visualized using FigTree v. 1.4.4. or iTOL (<https://itol.embl.de>), and trees for tungstoenzymes were edited with Inkscape v. 0.92.4.

In addition to phylogenetic reconstruction for genes interrogated, GraftM v. 0.11.1^37^ was used, with hmmsearch v. 3.2.1^38^ to identify the genes from the genomes that matched the constructed reference database. Maximum-likelihood placement of those sequences into the reference tree was then conducted.

***Supplementary Note 4: Taxonomy of the family Wolframiiraptoraceae***

Although previous studies have proposed that the Aigarchaeota may represent a novel phylum-level lineage^39^, recent increases in the number of genome sequences coupled with robust phylogenomics suggest this group belongs to the class *Nitrososphaeria* in the phylum *Thermoproteota*. Thus, based on the data presented here and other available data, we conservatively consider the Aigarchaeota synonymous to the order-level group *Caldarchaeales* as proposed by the Genome Taxonomy Database (GTDB; ref. ^40^). In this regard, we investigated one of the putative family-level lineages (f__NZ13-MGT in the GTDB) within the order *Caldarchaeales*.

Our evolutionary hypothesis recovered the lineage NZ13-MGT as monophyletic and exclusive (Figs. 5 and Supplementary Fig. 10), and fully supported, based on traditional bootstrap analysis, the placement of this lineage in the order *Caldarchaeales*. Although limited public genome data is available for this order, a robust phylogeny was reconstructed placing the lineage NZ13-MG1 as sister-taxon to the family *Caldarchaeaceae*, containing *Caldarchaeum subterraneum* and an unidentified genus-level group HRBIN01, with 99 % bootstrap support. Based on this phylogeny, *Calditenuaceae* represents the family splitting off at the most basal node within the order. We propose the family *Wolframiiraptoraceae* to accommodate the lineage NZ13-MG1. Within *Wolframiiraptoraceae*, four fully supported clusters were recovered, corresponding to previously hypothesized genus-level groups^41^.

Clear genomic and geographic separation is observed among species within the family. This is also supported by overall-genome-relatedness-indices. To assess conspecificity of species within the family, ANI values among members of the same genus, based on phylogenomic analysis and RED, employed in GTDB-Tk, were calculated. All pairwise ANI values within each of the eight species with several genome representatives available were above 97 %, while all comparisons among species of the same genus resulted in ANI values below 90 % (Supplementary Data 4).

To place the family *Wolframiiraptoraceae* into phylogenetic context, a domain-based phylogeny, including representatives of all archaeal phyla based on the representative taxon set established previously^25^ was constructed (Supplementary Fig. 10). All phylum-level groups were recovered as monophyletic, and most putative class-level groups within the *Thermoproteota* were also recovered as monophyletic. Within this phylogeny, the *Korarchaeia* split off at the most basal node within the phylum, with the classes *Thermoproteia* (formerly *Crenarchaeota*), *Geoarchaeia, Methanomethylicia* (including the order *Nezhaarchaeales*), and *Marsarchaeia* grouping together, while *Bathyarchaeia* grouped with the *Nitrososphaeria* (containing the *Caldarchaeales*). Within the class *Nitrososphaeria*, the *Caldarchaeales* split off at the most basal node within the class.

All names proposed for novel taxonomic ranks in this study has been registered and approved under the SeqCode (https://disc-genomics.uibk.ac.at/seqcode/), in registry list seqco.de/r:slp2ijs4.

***Supplementary Note 5: Physiology and evolution of physiological traits in Wolframiiraptoraceae***

Although *Wolframiiraptor* appears to be a strict anaerobe, some members of the genera *Terraquivivens*, *Geocrenenecus* and *Benthortus* may be facultative anaerobes based on the presence of genes encoding cytochrome *c* oxidase and cytochrome *bd*-1 ubiquinol oxidase in many of the MAGs (Supplementary Fig. 11a, b). The presence of genes encoding sulfide:quinone oxidoreductase (Sqr) in all genera of the family except *Wolframiiraptor* further suggests that some species may be capable of sulfide detoxification and/or chemolithotrophic sulfide oxidation under anoxic or oxic conditions (Supplementary Fig. 11a, b). *Geocrenenecus* MAGs also encode homologs of dissimilatory sulfite reductase (DsrAB) complexes and several heterodisulfide reductase (Hdr) complexes, one of which was consistently adjacent to a gene cluster encoding an F420-non-reducing hydrogenase complex (MvhADG). A Mvh:Hdr complex could potentially couple H_2_ oxidation as a source of electrons for sulfite reduction through DsrC. *Geocrenenecus* and *Benthortus* also encode homologs of putative aerobic Form II CO dehydrogenase complexes (CoxLMS) and cytochrome c oxidases, which could serve as an energy supplement for aerobic growth.

In-depth phylogenetic analyses of tungsten-associated enzymes were conducted to reconstruct the phylogenetic relationships and correlate evolutionary events with results obtained from the reconstruction of ancestral character states and reconciliations with the species tree. Although the presence of ModA, TupA and WtpA-like sequences in members of *Wolframiiraptoraceae* were all predicted with high probability as gene gains in the ancestral character state reconstruction (ACR) at specific nodes within the phylogeny of the family, sequences coding for the TupA and WtpA-like subunits are also found in other members of the *Thermoproteota* ( Supplementary Fig. 12). This distribution, particularly with the TupA subunit, indicated that we cannot exclude the possibility that these transporters may have arisen earlier in the evolution of the phylum, with subsequent losses occurring in the lineages lacking these genes, like in the case of the *Nitrososphaerales*. However, reconciliation analyses showed likely independent lateral acquisitions from within the *Thermoproteota* in *Wolframiiraptor* (and subsequent transfer from *W. gerlachensis* to *W. sinensis*), *Terraquivivens* (with subsequent transfer from *T. yellowstonensis* to the ancestor of *T. ruidianensis* and *T. tikiterensis*), and *Benthortus.* In the case of ModA, no close relatives within the Archaea were identified with these analyses, which indicates that the Mod ABC transporter in *T. tikiterensis* was horizontally acquired (supported by both analyses), likely from the *Proteobacteria*, although more detailed analyses regarding this transporter would be required to confirm this. The novel TupA-like sequence cluster identified here was only identified from some members of *Caldarchaeales*, with no known orthologs found in public databases with the approach employed here.

W-dependent ferredoxin oxidoreductases identified from members of *Wolframiiraptoraceae* were difficult to assign functions to, despite the phylogenetic and structural modeling approaches employed. This is due to most of these sequences being recovered as distinct monophyletic clusters, separate from known tungsten-dependent ferredoxin oxidoreductases. In order to recognize the distinct clusters, high branch support (> 0.8 SH-aLRT and > 90% Ultrafast bootstrap) at nodes recovering reference sequences as grouping close to *Wolframiiraptoraceae* sequences were used, e.g., the homologs in *Wolframiiraptoraceae* that clusters with high support with known FOR sequences were denoted as the FOR-like lineage (Supplementary Data 3, Supplementary Fig. 7).

The closest relatives to *Wolframiiraptoraceae* sequences, denoted as the AOR-like 1 cluster, were several members of the Terrabacteria. These included some members of the *Actinobacterota*, *Chloroflexota*, *Deinococcota* and numerous *Firmicutes* (Supplementary Fig. 8). Due to the lack of close relatives from the *Thermoproteota* for these sequences, acquisition of this gene via horizontal gene transfer (HGT), as predicted during reconstruction of ancestral character states as well as during reconciliation analyses, is plausible. As such, we propose that the AOR-like 1 lineage of homologs were present in the last common ancestor (LCA) of the family *Wolframiiraptoraceae*. Based on the data presented here, the likely genetic source for these sequences within the family may be members of the Terrabacteria.

For the sequences denoted as the AOR-like 2 cluster, several sequences from members of *Bathyarchaeia* were closely related to those identified from *Wolframiiraptoraceae* (Supplementary Fig. 8). Orthologs for this cluster were also only identified in members of the genera *Geocrenenecus* and *Wolframiiraptor*. With ancestral character state reconstruction, analyses predicted that the AOR-like 2 homologs were present in the LCA to the genera *Geocrenenecus* and *Wolframiiraptor*. In contrast, reconciliation analyses predicted a likely unknown origin for this cluster, with independent lateral transfers from this source to the LCA of the genus *Geocrenenecus*, bacteria and other *Thermoproteota*, with a subsequent transfer from the *Thermoproteota* to the LCA of the genus *Wolframiiraptor*. In this case, the very limited distribution of these sequences within the *Thermoproteota* may be indicative of an HGT event from the *Wolframiiraptoraceae* (some members of the family are splitting off at the most basal node in this cluster) to the *Bathyarchaeia*. There are also a few sequences identified as members of the *Chloroflexota* and *Nanoarchaeota* in this cluster, although the presence of these sequences in *Wolframiiraptoraceae* members are more numerous and diverse than those observed in any of these groups, indicating that members of the *Wolframiiraptoraceae* may represent the source of this homolog.

The cluster denoted as AOR-like 3 sequences grouped closely to those recovered from several *Thermoproteota* from the classes *Bathyarchaeia* and *Thermoproteia*, along with members of the *Firmicutes* and *Hadesarchaeota* (Supplementary Fig. 8). The presence of this sequence in several other *Thermoproteota* may be indicative of this sequence having been acquired earlier within the phylum, with subsequent losses occurring in lineages lacking orthologs of this cluster. However, the limited distribution within the phylum (with very few orthologs detected), coupled with the absence of these sequences from the genomes of other members of the *Caldarchaeales* appears to support the hypothesis that this gene was gained in an ancestor of *Wolframiiraptoraceae*, as indicated by the ACR, or through independent acquisitions in *Terraquivivens* and *Wolframiiraptor* from the *Thermoproteota*, although the evolutionary history of this gene may be complex with several horizontal transfers occurring between species in a genus, and even between domains.

All species belonging to *Wolframiiraptoraceae* were predicted to code for a single copy of putative tungsten-dependent ferredoxin oxidoreductase denoted as the FOR-like lineage (Supplementary Fig. 7). These sequences grouped closest to known formaldehyde ferredoxin oxidoreductases (FOR) found in numerous other members of the *Thermoproteota*, with high support (Supplementary Fig. 8). The vast majority of sequences in the neighboring lineage belonged to the classes *Bathyarchaeia*, *Korarchaeia* and *Thermoproteia*. Additionally, some sequences from members of the *Acidobacteriota*, *Bipolaricaulota*, *Desulfobacterota*, *Methanobacteriota*, and *Thermotogota* were nested within this cluster, possibly indicative of HGT events to members of other phyla. In this case, the putative functional assignment of these sequences as an FOR was also supported by structural modeling analyses, where representative sequences of this cluster were grouped with other functionally characterized FOR sequences (Supplementary Fig. 7). Due to the universal presence of the FOR-like homolog, the ACR suggest that this homolog was present in the LCA to the family, and this hypothesis is supported by the recovery of this cluster of sequences from the *Wolframiiraptoraceae* as monophyletic. In contrast, reconciliation analyses predict the less likely independent acquisition of these homologs in each of the genera. Based on this data, we propose that the FOR-like homolog was gained by the LCA of the family with rampant lateral exchange among members within the family.

Another cluster with a putative functional assignment based on the phylogeny and the structural modeling results was denoted as the GAPOR-like lineage (Supplementary Fig. 7). Although sequences of the archaeal glyceraldehyde-3-phosphate ferredoxin oxidoreductase (GAPOR) typified by the *Pyrococcus furiosus* GAPOR were included in the analyses, this sequence belonged to a distinct cluster containing only members of the *Methanobacteriota*, while a cluster containing numerous archaeal sequences including those from several classes of the *Thermoproteota* were recovered as monophyletic with high support with a bacterial cluster containing functionally characterized *Caldicellulosiruptor bescii* GAPOR sequence (referred to as GOR in Scott et al., 2019, ref. ^29^). The wide distribution of these GAPOR homologs within the *Thermoproteota* (including classes *Bathyarchaeia*, *Korarchaeia*, *Methanomethylicia*, *Nitrososphaeria* and *Thermoproteia*), along with those recovered from *Geocrenenecus* and *Wolframiiraptor* suggest that the gene encoding these proteins may represent an ancestral gene within the *Thermoproteota*, with gene losses occurring in other families with representation in *Caldarchaeales*, and the *Nitrososphaerales* (Supplementary Fig. 8), as predicted by the ACR. However, independent acquisitions with vertical descent were predicted as the most likely evolutionary hypothesis for this gene with reconciliation analyses.

Several lineages within the tungsten-dependent ferredoxin oxidoreductases were denoted as unknown lineages (Supplementary Fig. 7). This was true for multiple copies of these genes found in members of *Geocrenenecus* and *Terraquivivens*. In all three unknown lineages, all sequences were recovered from the *Wolframiiraptoraceae*, i.e., these lineages represent unique and novel sequences that have no detected orthologs in public databases.

In order to assess the reliability of annotations for genes related to the metabolism of these organisms, and to interrogate evolutionary events relating to these genes (as indicated by ACR), single gene trees (File S4), coupled with maximum-likelihood placement of sequences on reference phylogenies (Supplementary Data 5), were performed with the peptide sequences for genes of interest. Overall, annotation results were consistent with results obtained for phylogenies and phylogenetic placement analyses, with the exception of several genes annotated with multiple KO’s of interest. The genes interrogated in this way were *abfD* (K14534), *coxA* (K02274), *coxB* (K02275), *coxC* (K02276), *coxAC* (K15408), *coxL* (K03520), *cydA* (K00425), *dsrA* (K11180), *dsrB* (K11181), *mcmA1* (K01848), *mcmA2* (K01849), *nirK* (K00368), *nosZ* (K00376) and *sqr* (K17218).

Two genes encoding 4-hydroxybutyryl-CoA dehydratase (*abfD*) were identified with EggNOG, from the genome of *T. tikiterensis*, with no other representatives identified in the family *Wolframiiraptoraceae*. Upon closer inspection, it was noticed that one gene encoded a markedly shorter product than the other, and that the genes were located directly adjacent to one another on the genome, with a combined length closer to what is typically observed for *abfD*. Because of this, the genomic region combining both genes (NDWU01000005.1 from position 29,293 to 30,759; WP_011344625.1) was compared to the alignment of the reference sequences, and upon verification that alignment occurred over the full length of the sequence, this combined gene was used for phylogenetic analysis. Whether the gene within the genome of the organism is indeed interrupted by a premature stop codon, either representing two genes containing separate domains or representing a pseudogene, or whether erroneous base-calling occurred during sequencing would require further genomic representation of this species. Interestingly though, despite the frequent presence of *abfD* in members of the *Nitrososphaerales* (e.g., see File S4) who are the closest phylogenetic neighbors to *Caldarchaeales*, the gene in *T. tikiterensis* grouped most closely to some members of the *Chloroflexota* (*Anaerolineae*), *Desulfobacterota* (*Desulfosarcina* and *Desulfarculus*)*, Firmicutes* (*Carboxydothermus* and *Desulfosporosinus*), and *Halobacteriota* (*Archaeoglobus* and *Geoglobus*) (File S4), supporting a lateral acquisition in *T. tikiterensis*, as predicted by the ACR.

Several members of *Wolframiiraptoraceae* contain genes encoding the subunits for cytochrome C oxidase [*coxA*, *coxB*, *coxC*, *coxAC* (File S4)], particularly in the genera *Geocrenenecus* and *Benthortus* (Supplementary Fig. 11), with most members of *G. dongiae* and *G. huangii* encoding both *coxA* and *coxB*, and *B. lauensis* likely encoding multiple copies of *coxA*, *coxB* and *coxAC*. Sequences of both *coxA* and *coxB* for members of *G. dongiae* and *G. huangii* grouped monophyletically, followed by sequences for *B. lauensis*, with the closest relatives, recovered with high branch support including other members of the *Caldarchaeales* and the *Thermoproteota*, with some sequences grouping closely to the reference sequences of *Aeropyrum pernix*. One gene annotated as *coxA* for *B. lauensis* and *G. dongiae* JZ-1.bins.49.201705 each, grouped together, but separate from all other *coxA* sequences, and are likely *coxAC* genes based on GraftM results and additional phylogenetics (Supplementary Data 5), while another sequence annotated as *coxA* for *G. dongiae* JZ-1.bins.49.201705 (NODE_5_length_130183_cov_3.79904_92) showed markedly lower mean posterior probability (acc) for the reliability of the alignment than other *coxA* sequences based on the GraftM results (Supplementary Data 5). ACR analysis indicated the likely presence of these genes within an ancestor of the *Caldarchaeales* and *Nitrososphaerales* as a whole, followed by subsequent losses throughout the family, specifically in *Wolframiiraptor* and *Terraquivivens*, which, overall, appears to be congruent with the clustering of these sequences with members of the *Thermoproteota*. However, the lack in recovering consistent monophyletic groups among the different subunits would suggest that sufficient sequence divergence among these genes in this family has occurred as to prove difficult to obtain robust alignments, and further analyses would be required to confirm this hypothesis.

Based on reconstruction of ancestral states, *coxL* (encoding the carbon-monoxide dehydrogenase large subunit) may have been present in an ancestor of the *Caldarchaeales*, and this is supported by the phylogeny as all sequences from this order group monophyletically. This phylogenetic pattern within the family is consistent with vertical descent of this gene. However, these results are unclear regarding whether this represents an ancestral gene in the phylum *Thermoproteota*, or at least the class *Nitrososphaeria*, with subsequent losses in the *Nitrososphaerales* (as the ancestral reconstruction suggests), or whether a horizontal ancestral acquisition coupled with vertical descent within the family occurred, as several closest members include additional members of the *Thermoproteota*, particularly the *Bathyarchaeia*, as well as several bacterial phyla.

Several genomes in *Wolframiiraptoraceae* were annotated as containing multiple copies of *cydA*. This gene encodes the A subunit of cytochrome *bd* oxygen reductase and can either associate with the B subunit encoded by *cydB*, or with an A’ subunit encoded by *cydA’*, found exclusively in Archaea^42^. The CydAA’ cytochrome *bd* oxygen reductase is prevalent in the *Thermoproteia*, whereas several *Nitrososphaeria* encode different subunit genes to those associated with CydAA’^42^. Both *Wolframiiraptoraceae* clusters recovered in the CydA phylogeny grouped with high support with other archaeal sequences (Supplementary Fig. 18), including those from other *Thermoproteia* encoding CydAA’^42^, and our data suggest that those members of *Wolframiiraptoraceae* that encode a cytochrome *bd* oxygen reductase likely also encode the CydAA’ isoform. As such, the CydAA’ may represent an ancestral *Thermoproteota* trait, with replacement of CydA’ subunits in the *Nitrososphaerales*, however, ACR predicts likely independent acquisition events (gains) in the lineages within *Wolframiiraptoraceae* that encode the proteins.

Only members of *Geocrenenecus* were predicted to contain the genes encoding the α- and β- subunits of dissimilatory sulfite reductase [*dsrA* and *dsrB*], with a likely acquisition event occurring in the ancestor to the genus, coupled with vertical descent. This is supported by the recovery of a monophyletic group containing all *Geocrenenecus* sequences. These genes are located adjacent to one another on the genomes, and would have been transferred together in a single event. The likely source of the HGT event is however unclear, as the closest relatives between the two phylogenies are not in agreement. For DsrA, the closest relatives included several bacterial phyla, with numerous members of the *Actinobacteriota* and *Firmicutes*, while those for DsrB were members of the class *Thermoproteia*.

Of all *Wolframiiraptoraceae* members, only those genomes belonging to *Benthortus lauensis* and *Geocrenenecus arthurdayi* were predicted to encode methylmalonyl-CoA mutase [*mcmA*1 and *mcmA*2]. Several members of the *Nitrososphaerales* and other members of the *Thermoproteales* also encode these genes, however, interestingly these different orders in the *Thermoproteota* are not recovered as monophyletic, which may indicate independent acquisition events of these two genes in these lineages. This is also observed within the *Wolframiiraptoraceae*, where *B. lauensis* clustered with the sequences from the *Nitrososphaerales,* potentially pointing to a marine-associated HGT event, while those from *G. arthurdayi* grouped with members of the *Thermoproteales*. This, together with the ACR for these genes support independent acquisitions for those members of the family that encode this gene.

Two genes involved in nitrogen metabolism, *nirK* (nitrite reductase) and *nosZ* (nitrous-oxide reductase), were also interrogated. Several copies of *nirK* in *B. lauensis* were predicted, although these sequences only had e-value scores ranging between 1e^-05^ to 1e^-25^ with posterior probability alignment scores of 0.76 to 0.93. The identity of all of these genes are thus not robustly supported as *nirK*, but all sequences grouped with members of the *Nitrososphaerales*, similar to what would be expected for genes passed along by vertical descent with subsequent duplication events and gene losses in those taxa lacking this gene. A single genome in *G. dongiae* (JZ-1.bins.49.201705) was predicted to encode NosZ, while NosZ was also annotated for *B. lauensis*. Both sequences were well supported with the GraftM analysis, and grouped closely to some other *Thermoproteota* NosZ sequences in the phylogeny with good support. ACR analyses however, predicted acquisition events for these genes, indicating that lateral transfer from other *Thermoproteota* may be possible.

All species in *Terraquivivens* and *Geocrenenecus* (except *G. arthurdayi*) were predicted to possess genes encoding sulfide quinone oxidoreductase (Sqr). All Sqr sequences from these members of *Wolframiiraptoraceae* were recovered as a monophyletic group with a single copy of this gene predicted in *B. lauensis* in a distinct grouping from the rest of the family. *B. lauensis* grouped with *Nitrosocaldus cavascurensis* (A0A2K5ANR2), *Hyperthermus butylicus* (A2BK70), and *Aeropyrum pernix* (Q9YD69), along with other *Thermoproteia*, while those recovered for *Terraquivivens* and *Geocrenenecus* group most closely with sequences from several bacterial phyla. The ancestral state reconstruction predicted sulfide oxidation or detoxification to represent an ancestral trait shared by *Caldarchaeales* along with *Nitrososphaerales*, however, whether independent horizontal acquisitions were in fact the cause for the current observed distribution, or whether a more complicated evolutionary history underlies these genes remain to be seen.

***Supplementary Note 6: Protologues***

**Description of the family *Candidatus* Wolframiiraptoraceae**

Wolf.ra.mi.i.rap.to.ra'ce.ae N.L. fem. n. *Wolframiiraptor*, type genus of the family; L. fem. pl. suff. *-aceae*, ending to denote a family; N.L. fem. pl. n. *Wolframiiraptoraceae*, family of the candidate genus *Wolframiiraptor*

Members of this family are associated with thermal aquatic environments, and have been identified from geothermal springs in China, New Zealand and the USA, and a marine hydrothermal vent in the Western Pacific. Phylogenomic inference robustly recovers the genomes of these organisms as a well-supported monophyletic lineage within the order *Caldarchaeales*, and delineation of these taxa as a family is supported by Relative Evolutionary Divergence (RED) and Average Amino Acid Identity (AAI). AAI values among designated type genomes for species in this family range between 65 and 85 % within proposed genera, and between 49 and 57 % among members of different genera. The distribution of genes required for oxidative phosphorylation indicate that members of the family may either be strict or facultative anaerobes. Oxidation or reduction of sulfur compounds may also occur in some members of the family, but this trait is not conserved for all genera. Several putative tungsten-dependent ferredoxin oxidoreductases, specifically aldehyde ferredoxin oxidoreductases (AORs), formaldehyde ferredoxin oxidoreductases (FORs) and glyceraldehyde-3-phosphate ferredoxin oxidoreductases (GAPORs) are encoded by genomes belonging to this family.

The nomenclatural type for the family is the genus *Wolframiiraptor*.

**Description of the genus *Candidatus* Benthortus**

Benth'or.tus Gr. fem. n. *benthos*, deep water; L. pass. part. *ortus*, risen; N.L. masc. n. *Benthortus*, risen from deep water

The genome of the current sole member of this genus was recovered from metagenomic sequencing data of hydrothermal vent communities present in the Lau Basin in the Western Pacific Ocean. AAI values among this genus and closely related genera range between 50 and 57 %. Ancestral state reconstruction analysis indicated likely acquisitions of multiple copies of genes encoding methylmalonyl-CoA mutase (McmA1/2), NO-forming nitrite reductase (NirK), and cytochrome C oxidase subunit III (CoxC), and one copy of genes encoding nitrous-oxide reductase (NosZ) and aerobic carbon-monoxide dehydrogenase large subunit (CoxL), each. The tungsten (Tup) transporter substrate-binding subunit A is encoded by the genome representative of this genus, and the AOR- and FOR-like encoding genes conserved within the family are also present in this genus. The presence of several cytochrome C oxidase subunits and the aerobic carbon-monoxide dehydrogenase large subunit indicate likely capacity for aerobic respiration within the genus. A sulfide:quinone oxidoreductase (*sqr*) gene is present in the genome and also indicates likely sulfide oxidation within the genus. This genus is supported as distinct and exclusive based on phylogenomics, AAI and RED, and is part of the family *Wolframiiraptoraceae*.

The nomenclatural type for the genus is *Benthortus lauensis*.

**Description of *Candidatus* Benthortus lauensis**

lau.en'sis N.L. masc. adj. *lauensis*, of Lau, referring to the Lau Basin in the Western Pacific, where this organism was identified from

The MAG of this organism was recovered from metagenomic sequencing data of a deep-water hydrothermal sulfide deposit sample taken from the Lau Basin, in the Western Pacific. The binned genome of this organism is 1,470,116 bp in size, and consists of 112 contigs, with a G+C content of 54.8 %. CheckM-based quality assessment indicates a completeness of 94.0 % and a contamination estimate of 2.91 %. Phylogenomic reconstruction based on 122 conserved archaeal genes place this taxon in the family *Wolframiraptoraceae*. Only two putative tungsten-dependent AORs were predicted within the genome of this organism.

The nomenclatural type for the species is the genome T2-175 (available under the GenBank assembly accession number: GCA_021650775.1, NCBI BioProject ID: PRJNA495050) and was identified from a hydrothermal sulfide deposit sample in the Lau Basin, Western Pacific.

**Description of the genus *Candidatus* Geocrenenecus**

Ge.o.cren.en.e’cus Gr. fem. n. *ge*, the earth; Gr. fem. n. *krene*, a spring; N.L. masc. n. *enecus*, inhabitant; N.L. masc. n. *Geocrenenecus*, inhabitant of a spring of the earth

Genomes of members of this genus have been recovered from metagenomic sequencing of samples from thermal springs in Yellowstone National Park, USA, and the Rehai and Ruidian geothermal fields, Tengchong, China. AAI values among different species in the genus range between 67 and 78 %. Ancestral reconstruction of character states indicated likely losses of the tungstate (Tup) transporter system, along with loss of the tungsten-dependent AOR-like encoding gene found in other members of the family. Likely gains within the genus were genes encoding dissimilatory sulfite reductase subunits (*dsrA/B*) along with sulfide:quinone oxidoreductase (*sqr*), which suggest pathways involved in sulfur metabolism, specifically sulfur or sulfide oxidation, and sulfite reduction within the genus. No homologs to known tungstate or molybdate transporters were identified from any of the MAGs belonging to the genus. Several putative tungsten-dependent ferredoxin oxidoreductases were identified as conserved within the genus, which included one AOR-like and one FOR-like encoding gene, respectively, conserved within the family, one AOR-like encoding gene conserved within this genus and its closest phylogenetic relative, *Wolframiiraptor,* and an unknown protein shared with members of *Terraquivivens*. Based on the presence of cytochrome C oxidase subunits, some members of this genus may be capable of aerobic respiration. Phylogenomics, AAI and RED values, support the delineation of this taxon as a genus.

The nomenclatural type for the genus is *Geocrenenecus dongiae*.

**Description of *Candidatus* Geocrenenecus arthurdayi**

ar.thur.day’i N.L. gen. n. *arthurdayi*, of Arthur Day, named for the American geophysicist and volcanologist Dr. Arthur L. Day (1869-1960), for his seminal work in Earth sciences, particularly relating to the hot springs in Yellowstone National Park, USA

MAGs identified as belonging to this species were recovered from metagenomes sampled from two thermal springs in Yellowstone National Park, USA. The six MAGs for this species range between 1,530,521 bp and 1,985,835 bp, in 82-223 contigs, with a G+C content of 38.4-39.4 %. Completeness estimates were between 96.1 and 97.8 %, with 0 % contamination, based on CheckM. Phylogenomic inference based on the ar122 conserved archaeal marker set placed the species within the genus *Geocrenenecus*, in the family *Wolframiiraptoraceae*. All genomes of this species were considered conspecific based on Average Nucleotide Identity (ANI), with pairwise values >99 %. Comparisons against the closely related members of the genus resulted in pairwise values between 75 and 78 %. No homologs to known or putative tungstate or molybdate transporters were identified from any of the genomes belonging to this species. Unlike other members of the genus, no GAPOR-like tungsten-dependent ferredoxin oxidoreductase was identified in this species. One genome encoded an unknown oxidoreductase. All genomes belonging to this species encode methylmalonyl-CoA mutase (McmA1/2), indicative of potential propionate metabolism. Unlike other species in the genus, the genomes of this species lack genes encoding cytochrome c oxidase subunits, but contain genes encoding cytochrome *bd* ubiquinol oxidase subunit I (*cydA*), and the aerobic carbon-monoxide dehydrogenase large subunit (*coxL*). The genomes within this species does also encode a homolog of *arxA*, suggesting potential respiratory arsenate reduction.

The nomenclatural type for the species is the genome LGB-05.bin.6 (submitted under the NCBI WGS accession number: JAKCEX000000000, NCBI BioProject ID: PRJNA791658), recovered from metagenomic sequence data of a sediment sample from a hot spring in Yellowstone National Park, USA.

**Description of *Candidatus* Geocrenenecus dongiae**

dong'i.ae N.L. gen. n. *dongiae* named for the Chinese microbiologist, Prof. Xiuzhu Dong (1958-), for her contributions to the study of ecology and taxonomic classification of extremophiles

Fifteen MAGs representing this species were recovered from metagenomic sequencing efforts of thermal springs in the Rehai and Ruidian geothermal fields, Tengchong, China. The genomes ranged in size from 1,322,968 bp to 1,558,478 bp, in 21 to 188 contigs, and ranged in G+C content between 37.4 and 37.6 %. The genomes were estimated to be between 90.9 and 99.1 % complete, with limited contamination (0-0.97 %) based on CheckM. Phylogenomic analysis of 122 conserved archaeal markers robustly placed this taxon in the novel genus *Geocrenenecus*, in the family *Wolframiiraptoraceae*. ANI comparisons within the species resulted in values above 99 %, while all comparisons to closest relatives were below 90 %. Similar to other members of the genus, no homologs to known tungstate or molybdate transporters were identified from members of this species, although one genome contained genes that were identified as *tupA*-like*,* as well as a homolog to the proposed *wtpA/modA*-like genes identified in *Pyrobaculum* species previously. This genome also encoded *nirK*, *nosZ* and *coxAC*, while *mcmA1* and *mcmA2* were identified in two genomes, however, all other members identified in this study belonging to this species lacked these genes. In addition to the several tungsten-dependent ferredoxin oxidoreductases conserved within the genus, all members of this species encode a GAPOR-like enzyme. The genomes of most members in the species encode the genes for cytochrome C oxidase subunits (*coxA/B*), cytochrome *bd* ubiquinol oxidase subunit I (*cydA*), and the aerobic carbon-monoxide dehydrogenase large subunit (*coxL*), which may indicate that members of this species can perform aerobic respiration.

The nomenclatural type for this species is the genome ZZQ.bins.17.201803 (available under the eLMSG assembly accession number: LMSG_G000004221.1, NCBI BioProject ID: PRJNA807863) and was recovered from metagenomic sequencing efforts of a sediment sample taken from the thermal spring Zhenzhuquan, in Tengchong, China.

**Description of *Candidatus* Geocrenenecus huangii**

huang'i.i N.L. gen. n. *huangii*, of Huang, named for the Chinese microbiologist, Prof. Li Huang (1958-), for his contributions to the study of virus ecology and taxonomic classification in hot springs

Twelve MAGs for this organism were recovered from metagenomes of thermal springs in the Rehai and Ruidian geothermal fields, Tengchong, Yunnan, China. The binned MAGs ranged between 1,361,286 bp and 1,697,407 bp, in 21 to 174 contigs, with a G+C content of 40.5 to 40.7 %. Genome completeness estimates were between 91.3 and 98.1 %, with contamination estimated at 0.97 to 1.46 % based on CheckM. Robust phylogenomic analysis based on 122 archaeal markers places this taxon in the genus *Geocrenenecus*, in the family *Wolframiiraptoraceae*. Intraspecific ANI values all range between 99.3 and 100 %, while all other comparisons to other members within the genus range between 75 and 90 %. Like other members of the genus, several AORs were identified in the genomes of this species, with a GAPOR-like encoding gene in all members of the species. The presence of genes encoding some cytochrome C oxidase subunits (*coxA/B*), cytochrome *bd* ubiquinol oxidase subunit I (*cydA*), and the aerobic carbon-monoxide dehydrogenase large subunit (*coxL*), suggest that members of this species may be capable of aerobic respiration.

The nomenclatural type for the species is the MAG ZZQ.bins.13.201803 (available under the eLMSG assembly accession: LMSG_G000004232.1, NCBI BioProject: PRJNA807863), identified from metagenomic sequencing of a sediment sample taken from Zhenzhuquan spring, Tengchong, China.

**Description of the genus *Candidatus* Terraquivivens**

Ter.ra.qui'vi.vens L. fem. n. *terra*, the earth; L. fem. n. *aqua*, water; N.L. pres. part. *vivens*, living; N.L. fem. n. *Terraquivivens*, living in water from the earth

Genomes of members of this genus were recovered from metagenomic sequencing of samples obtained from geothermal springs from China, New Zealand and USA. AAI values among members of different species within the genus range between 78 and 86 %. Based on ancestral state reconstruction analysis, likely losses of cytochrome C oxidase and aerobic carbon monoxide dehydrogenase subunit encoding genes occurred in an ancestral population to the genus, indicating that members of this genus are likely anaerobes. Although no *in vitro* evidence of tungsten utilization or dependence for this organism is available, the presence of genes encoding the tungstate ABC transporter (*tupABC*) is conserved within the genus, which suggest the ability to take up tungstate from the environment. Of the tungsten-dependent AORs investigated in this study, several are conserved within the genus, but the types of aldehydes oxidized by these enzymes remain unclear. Sulfide oxidation is likely in these organisms as sulfide:quinone oxidoreductase (*sqr*)-encoding genes are mostly present in the genomes of these organisms. Delineation of this taxon as a genus-level group is supported by phylogenomics, AAI and RED values.

The nomenclatural type of the genus is the species *Terraquivivens tikiterensis*.

**Description of *Candidatus* Terraquivivens tikiterensis**

ti.ki.ter.en'sis N.L. fem. adj. *tikiterensis*, of Tikitere, referring to Tikitere, Rotorua, New Zealand, where this organism was identified from

The MAG of this organism was recovered from environmental sequencing of samples from Tikitere, Rotorua, New Zealand. The binned genome totals 1,536,389 bp and has a G+C content of 51.3 %. The genome completeness is estimated at 99.0 %, with very low (0.49 %) contamination based on CheckM. Comprehensive phylogenomic analysis of 120 archaeal marker genes places this taxon in the proposed candidate genus, *Terraquivivens*, in the proposed candidate family *Wolframiiraptoraceae*. ANI values between this taxon and other members of the genus fall well below species delineation guidelines (76-78 %), and along with geographic isolation of this taxon from close relatives, delineation as a novel species is supported. Along with the tungstate (Tup) ABC transporter subunits, the molybdate transporter (*modABC*) and an additional putative *wtpA/modA*-like gene, homologous to *modA*-like sequences in *Pyrobaculum* species, suggest versatility in uptake of either molybdate or tungstate by this species. Two gene fragments encoding 4-hydroxybutyryl-CoA dehydratase (*abfD*) together, have been identified from the genome, indicating potential autotrophic CO_2_ fixation through the oxygen-tolerant hydroxypropionate/hydroxybutyrate (HP/HB) cycle, while no other members of the family were found to possess these genes.

The proposed nomenclatural type of this taxon is the metagenome-assembled genome NZ13-MG1, deposited in GenBank under the assembly accession GCA_003056285.1 (NCBI BioProject: PRJNA381623), and was assembled from metagenomic sequence data of an enrichment culture from water and sediment collected from a thermal spring in Tikitere, Rotorua, New Zealand.

**Description of *Candidatus* Terraquivivens ruidianensis**

rui.di.an.en'sis N.L. fem. adj. *ruidianensis*, of Ruidian, referring to the Ruidian geothermal fields, Yunnan, China, where these organisms were identified from

Two MAGs of this organism were recovered from environmental sequencing of samples from Jinze hot spring, in the Rehai geothermal field, Tengchong, Yunnan, China. The binned genomes are 1,273,812 bp in 8 contigs, and 1,298,744 bp in 50 contigs, respectively, and both genomes have a G+C content of 47.5 %. The genome completeness estimates for both are 98.5 %, with very low (0.97%) contamination based on CheckM. Phylogenomic analysis of the 120 archaeal marker genes in the ar122 dataset places this taxon in the proposed candidate genus, *Terraquivivens*, in the proposed candidate family *Wolframiiraptoraceae*. ANI values between the two genomes are 99.9 %, while values between this taxon and other members of the genus fall below species delineation guidelines (80-82 %).

The proposed nomenclatural type of this taxon is the metagenome-assembled genome JZ-2.bins.210.201808 (deposited in eLMSG under the assembly accession: LMSG_G000004233.1, and NCBI BioProject: PRJNA807863), and was assembled from metagenomic sequence data of a sediment sample taken at sample location two from the thermal spring Jinze, in the Ruidian geothermal field, Tengchong, Yunnan, China.

**Description of *Candidatus* Terraquivivens tengchongensis**

teng.chong.en'sis N.L. fem. adj. *tengchongensis*, of Tengchong, referring to Tengchong, China, where these organisms were identified from

Fifteen MAGs for this organism were recovered from several sampling locations in Diretiyanqu, Gumingquan, Jinze and Shuirebaozha thermal springs in the Rehai and Ruidian geothermal fields, Tengchong. Binned genomes for this species ranged between 1,094,328 bp and 1,283,151 bp, in 2 to 160 contigs, and ranged in G+C content from 44.8 to 45.3 %. Completeness estimates for these genomes were between 90.3 and 97.1 %, with contamination estimated at 0-2.91 %, based on CheckM. This species is placed in *Terraquivivens*, in the family *Wolframiiraptoraceae*, based on phylogenomic analysis using 122 archaeal marker genes. ANI values within the species range between 98 and 100 %, with all other values to the closest relatives being below species guidelines (79-85 %). In addition to the tungstate (Tup) ABC transporter detected in all members of the genus, the majority of the genomes for this species also encode a *wtpA/modA*-like protein that is homologous to *modA*-like sequences previously reported in *Pyrobaculum*. Most members of this species contain the gene *cydA* that encodes cytochrome *bd* ubiquinol oxidase subunit I, which appears to have been gained in only this species in the genus. Also, an unknown ferredoxin oxidoreductase, shared only by the sister species *Terraquivivens yellowstonensis*, is present in all genomes of this species studied here.

The nomenclatural type for this species is the metagenome-assembled genome GMQP.bins.8.201907, submitted under the eLMSG assembly accession LMSG_G000004244.1 (NCBI BioProject ID: PRJNA807863), and was assembled from metagenomic sequence data of a sediment sample from Gumingquan thermal spring, in Rehai geothermal field, Tengchong.

**Description of *Candidatus* Terraquivivens yellowstonensis**

yel.low.ston.en’sis N.L. fem. adj. yellowstonensis, of Yellowstone, referring to Yellowstone National Park, USA, where this organism was identified from

Seven MAGs for this species were identified from four different springs in Yellowstone National Park, USA. The genomes are between 1,073,374 bp and 1,275,530 bp, in 19 to 35 contigs, and have a G+C content between 45.9 and 46.1 %. Completeness and contamination estimates based on CheckM are between 94.17 and 97.08 %, and 0 to 1.94 %, respectively. Phylogenomics with the ar122 markers robustly place this species in the genus *Terraquivivens*, in the family *Wolframiiraptoraceae*. ANI values among genomes for this species are 98-100 %, with all other pairwise values compared to members of the genus below 85 %. The gene encoding Sqr (sulfide:quinone oxidoreductase) is present in some members of the species, however, most of the genomes lack this gene. All genomes belonging to the species lack an AOR-like encoding gene that is conserved within the rest of the family, and possess an unknown oxidoreductase that is shared with *Terraquivivens tengchongensis*, however, the substrates for these enzymes are still unclear.

The MAG LGB-05.bin.49 is designated as the nomenclatural type for the species (NCBI WGS accession number: JAKCEW000000000, NCBI BioProject ID: PRJNA791658), and was assembled from metagenomic sequence data from a sediment sample from a thermal spring, in Yellowstone National Park, USA.

**Description of the genus *Candidatus* Wolframiiraptor**

Wolf.ra.mi.i.rap’tor N.L. neut. n. *wolframium*, tungsten; L. masc. n. *raptor*, snatcher or thief; N.L. masc. n. *Wolframiiraptor*, snatcher of tungsten

Members of this genus have been identified from geothermal springs from the U.S. Great Basin, Yellowstone National Park, USA, and the Rehai and Ruidian geothermal fields, Tengchong, China. AAI values among genomes representing separate species within the genus range between 81 and 90 %. Based on ancestral state reconstruction analysis, likely losses of the genes encoding cytochrome C oxidase subunits, the aerobic carbon-monoxide dehydrogenase large subunit, and sulfide:quinone oxidoreductase (Sqr), indicate that members of this genus are likely strict anaerobes, and are also incapable of sulfide oxidation. Genomes of this genus encode a *tupA* subunit of the tungstate (Tup) ABC transporter, and contain several genes encoding for tungsten-dependent oxidoreductases, including three putative AOR-like, one FOR-like and one GAPOR-like proteins. This taxon is supported as a genus-level group by phylogenomics, AAI and RED.

The nomenclatural type for the genus is *Wolframiraptor gerlachensis*.

**Description of *Candidatus* Wolframiiraptor allenii**

al.len’i.i N.L. gen. n. *allenii*, of Allen, named for Dr. Eugene T. Allen (1864-1964), an American geochemist, for his pioneering work on the geochemistry of Yellowstone National Park, USA

Two MAGs representing this species were assembled from metagenomic sequence data determined from samples taken from thermal springs in Geyser Creek, Geyser Basin and the Lower Geyser Basin, Yellowstone National Park, USA. These genomes are 1,104,807 bp and 1,315,737 bp in size, 151 and 95 contigs, respectively, with a G+C content of 51.6 to 52.4 %. Completeness estimates for both genomes, as determined by CheckM, are 91.58 and 92.23 %, with 0 % contamination estimated. Phylogenomic analysis of 122 archaeal marker sequences places this species in the genus *Wolframiiraptor*, in the family *Wolframiiraptoraceae*. ANI values between these genomes are 99.0 %, while all other pairwise comparisons to closely related taxa are below 87 %.

The proposed nomenclatural type for the species is the MAG LGB-01.bin.22, available under the NCBI WGS assembly accession number (JAKCEV000000000), BioProject ID (PRJNA791658), recovered from a thermal spring in Lower Geyser Basin, in Yellowstone National Park, USA.

**Description of *Candidatus* Wolframiiraptor gerlachensis**

ger.lach.en'sis N.L. masc. adj. *gerlachensis*, of Gerlach, the region where Great Boiling Spring is located in Nevada, and this organism was obtained from

A MAG representing this species was recovered from metagenomic sequencing of a stable enrichment culture, established from an *in situ* corn stover enrichment from Great Boiling Spring, Nevada, USA. Enrichment and maintenance of this species within the mixed-culture community was optimal at an incubation temperature of 80 °C with lignocellulose and sugars, at circumneutral pH. This species is dependent on tungsten for growth, with significant decline in its abundance within the community without tungsten added to the growth medium. Additionally, several tungstoenzymes conserved within the genus were expressed at a higher rate during growth on corn stover, suggesting direct involvement of tungstoenzymes in complex carbohydrate metabolism. Cells of this organism showed significant isotope enrichment when grown on isotopically labeled xylose-amended medium, with limited isotope enrichment during growth on medium amended with amino acids, glucose, ribose, and starch, indicating preferential assimilation of xylose. The genome sequence for this organism is 1,277,965 bp in size, and consists of 27 contigs and has a G+C content of 52 %. Completeness is estimated at 98.06 % with 0.49 % contamination, as estimated with CheckM. ANI comparisons among this genome and those of closely related species were below 86 %, supporting the delineation of this taxon as unique and distinct to other species in the genus.

The genome A8, available under the GenBank assembly accession number GCA_021323375.2, NCBI BioProject ID: PRJNA780009, is the designated nomenclatural type for the species, and was recovered from an enrichment culture, established from an *in situ* enrichment from Great Boiling Spring, Nevada, USA.

**Description of *Candidatus* Wolframiiraptor sinensis**

sin.en’sis N.L. masc. adj. *sinensis*, of or pertaining to China, where the organisms were identified from

Sixteen MAGs representing this species were identified from thermal springs in the Rehai and Ruidian geothermal fields, Tengchong, China. These genomes ranged in size from 990,328 bp to 1,230,238 bp, in 10 to 90 contigs, with G+C content of 53.7-55.6 %. Genome completeness was estimated at 92-98.1 %, with 0-2 % contamination, as determined with CheckM. This species is placed in the genus *Wolframiiraptor*, in the family *Wolframiiraptoraceae*, based on phylogenomic analysis of 122 conserved archaeal marker sequences. ANI values among members of the species range between 97 and 100 %, with comparisons with other members of the genus resulting in values below 80 %.

The proposed nomenclatural type for the species is the MAG GMQP.bins.13.201907 (eLMSG accession number: LMSG_G000004251.1, NCBI BioProject ID: PRJNA807863), obtained from the geothermal spring Gumingquan, in Tengchong, China.

**Supplementary References**

1. Flores, G. E. et al. Inter-field variability in the microbial communities of hydrothermal vent deposits from a back-arc basin. *Geobiology* **10**, 333–346 (2012).
2. Colman, D. R., Lindsay, M. R., & Boyd, E. S. Mixing of meteoric and geothermal fluids supports hyperdiverse chemosynthetic hydrothermal communities. *Nature Communications* **10** (1), 681 (2019).
3. Kolmogorov, M., et al. metaFlye: scalable long-read metagenome assembly using repeat graphs. *Nature Methods* **17**, 1103–1110 (2020).
4. Nurk, S., Meleshko, D., Korobeynikov, A., & Pevzner, P. A. metaSPAdes: a new versatile metagenomic assembler. *Genome Research* **27** (5), 824-834 (2017).
5. Mikheenko, A., Saveliev, V., & Gurevich, A. MetaQUAST: evaluation of metagenome assemblies. *Bioinformatics* **32** (7), 1088-1090 (2016).
6. Kang, D. D., et al. MetaBAT 2: an adaptive binning algorithm for robust and efficient genome reconstruction from metagenome assemblies. *PeerJ* **7**,e7359 (2019).
7. Wu, Y.-W., Simmons, B. A., & Singer, S. W. MaxBin 2.0: an automated binning algorithm to recover genomes from multiple metagenomic datasets, *Bioinformatics* **32** (4), 605–607 (2016).
8. Arkin, A. P. *et al.* KBase: The United States Department of Energy Systems Biology Knowledgebase. *Nature Biotechnology* **36,** 566–569 (2018).
9. Parks, D. H., Imelfort, M., Skennerton, C. T., Hugenholtz, P. & Tyson, G. W. CheckM: assessing the quality of microbial genomes recovered from isolates, single cells, and metagenomes. *Genome Research* **25,** 1043–1055 (2015).
10. Chaumeil, P.-A., Mussig, A. J., Hugenholtz, P. & Parks, D. H. GTDB-Tk: a toolkit to classify genomes with the Genome Taxonomy Database. *Bioinformatics* **36** (6) 1925-1927 (2020).
11. Aziz, R. K. *et al.* The RAST Server: Rapid Annotations using Subsystems Technology. *BMC Genomics* **9,** 1–15 (2008).
12. Bolger, A. M., Lohse, M. & Usadel, B. Trimmomatic: a flexible trimmer for Illumina sequence data. *Bioinformatics* **30,** 2114–2120 (2014).
13. Li, D., Liu, C. M., Luo, R., Sadakane, K., & Lam, T. W. MEGAHIT: an ultra-fast single-node solution for large and complex metagenomics assembly via succinct de Bruijn graph. *Bioinformatics* **31** (10), 1674-1676 (2015).
14. Langmead, B., & Salzberg, S. Fast gapped-read alignment with Bowtie 2. *Nature Methods* **9**, 357–359 (2012).
15. Li, H., et al. The Sequence Alignment/Map format and SAMtools. *Bioinformatics* **25** (16), 2078-2079 (2019).
16. Kang, D. D, Froula, J., Egan, R., & Wang, Z. MetaBAT, an efficient tool for accurately reconstructing single genomes from complex microbial communities. *PeerJ* **3**, e1165 (2015).
17. Hua, Z. S., et al. Ecological roles of dominant and rare prokaryotes in acid mine drainage revealed by metagenomics and metatranscriptomics. *ISME Journal* **9**, 1280–1294 (2015). (10.1038/ismej.2014.212)
18. Bushnell, B. http://sourceforge.net/projects/bbmap/
19. Krueger, F. <https://www.bioinformatics.babraham.ac.uk/projects/trim_galore/>
20. Martin, M. Cutadapt removes adapter sequences from high-throughput sequencing reads. *EMBnet Journal* **17** (1), (2011).
21. Payne, D., et al. Geologic legacy spanning >90 years explains unique Yellowstone hot spring geochemistry and biodiversity. *Environmental Microbiology* **21** (11), 4180-4195 (2019).
22. Uritskiy, G. V., DiRuggiero, J., & Taylor, J. MetaWRAP-a flexible pipeline for genome-resolved metagenomic data analysis. *Microbiome* **6** (1), 158 (2018).
23. Li, H., & Durbin, R. Fast and accurate short read alignment with Burrows-Wheeler transform. *Bioinformatics* **25** (14), 1754-60 (2009).
24. Alneberg, J., et al. Binning metagenomic contigs by coverage and composition. *Nature Methods* **11**, 1144–1146 (2014).
25. Dombrowski, N., et al. Undinarchaeota illuminate DPANN phylogeny and the impact of gene transfer on archaeal evolution. Nature Communications 11, 3939 (2020).
26. Darriba, D., Taboada, G. L., Doallo, R., Bioinformatics, D. P. ProtTest 3: fast selection of best-fit models of protein evolution. FEMS Microbiology Reviews 27, 1164–1165 (2011).
27. Kück, P. & Longo, G. C. FASconCAT-G: extensive functions for multiple sequence alignment preparations concerning phylogenetic studies. Frontiers in Zoology 11, 1-8 (2014).
28. Nguyen, L.-T., Schmidt, H. A., Haeseler, A. V. & Minh, B. Q. IQ-TREE: A fast and effective stochastic algorithm for estimating maximum-likelihood phylogenies. Mol. Biol. Evol. 32, 268–274 (2014).
29. Minh, B. Q., Nguyen, M. A. T. & Haeseler, A. V. Ultrafast Approximation for Phylogenetic Bootstrap. *Mol Biol Evol* **30,** 1188–1195 (2013).
30. Guindon, S., et al. New algorithms and methods to estimate maximum-likelihood phylogenies: assessing the performance of PhyML 3.0. Systematic Biology 59 (3), 307-321 (2010).
31. Altschul, S. F., Gish, W., Miller, W., Myers, E. W. & Lipman, D. J. Basic local alignment search tool. *Journal of Molecular Biology* **215,** 403–410 (1990).
32. Bevers, L. E., Hagedoorn, P. L., Krijger, G. C., & Hagen, W. R. Tungsten transport protein A (WtpA) in *Pyrococcus furiosus*: the first member of a new class of tungstate and molybdate transporters. *Journal of Bacteriology* **188** (18), 6498-505 (2006).
33. Rozewicki, J., Li, S., Amada, K. M., Standley, D. M. & Katoh, K. MAFFT-DASH: integrated protein sequence and structural alignment. *Nucleic Acids Research* **14,** 249 (2019).
34. Katoh, K., Rozewicki, J. & Yamada, K. D. MAFFT online service: multiple sequence alignment, interactive sequence choice and visualization. *Briefings in Bioinformatics* **20,** 1160–1166 (2017).
35. Scott, I. M., et al. The thermophilic biomass-degrading bacterium *Caldicellulosiruptor bescii* utilizes two enzymes to oxidize glyceraldehyde 3-phosphate during glycolysis. *Journal of Biological Chemistry* **294** (25), 9995-10005 (2019).
36. Li,W., & Godzik, A. CD-Hit: a fast program for clustering and comparing large sets of protein or nucleotide sequences. *Bioinformatics* **22**(13), 1658-9 (2006).
37. Boyd, J. A., Woodcroft, B. J., & Tyson, G. W. GraftM: a tool for scalable, phylogenetically informed classification of genes within metagenomes. *Nucleic Acids Research* **46** (10), e59 (2018).
38. Mistry, J., Finn, R. D., Eddy, S. R., Bateman, A., & Punta, M. Challenges in homology search: HMMER3 and convergent evolution of coiled-coil regions, *Nucleic Acids Research* **41** (12), e121 (2013).
39. Nunoura, T. *et al.* Insights into the evolution of Archaea and eukaryotic protein modifier systems revealed by the genome of a novel archaeal group. *Nucleic Acids Research* **39,** 3204–3223 (2010).
40. Rinke, C. *et al*. A standardized archaeal taxonomy for the Genome Taxonomy Database. *Nature Microbiology* **6**, 946-959 (2021).
41. Hedlund, B. P. *et al.* Uncultivated thermophiles: current status and spotlight on ‘Aigarchaeota’. *Current Opinion in Microbiology* **25,** 136–145 (2015).
42. Murali, R., Gennis, R.B. & Hemp, J. Evolution of the cytochrome *bd* oxygen reductase superfamily and the function of CydAA’ in Archaea. *ISME J* (2021).
