# Supplementary material for: An essential role for tungsten in the ecology and evolution of a previously uncultivated lineage of anaerobic, thermophilic Archaea
Source: Nat Commun. 2022 Jun 30;13:3773. doi: 10.1038/s41467-022-31452-8 (PMC9246946; doi:10.1038/s41467-022-31452-8)
Supplement: Supplementary file 2 — Reporting Summary [file 41467_2022_31452_MOESM2_ESM.pdf]

Corresponding author(s): S. Buessecker, M. Palmer, J. A. Dodsworth

Last updated by author(s): May 14, 2022

Reporting Summary

Nature Portfolio wishes to improve the reproducibility of the work that we publish. This form provides structure for consistency and transparency in reporting. For further information on Nature Portfolio policies, see our Editorial Policies and the Editorial Policy Checklist.

Statistics

For all statistical analyses, confirm that the following items are present in the figure legend, table legend, main text, or Methods section.

n/a	Confirmed	
☐	☒	The exact sample size (*n*) for each experimental group/condition, given as a discrete number and unit of measurement
☐	☒	A statement on whether measurements were taken from distinct samples or whether the same sample was measured repeatedly
☐	☒	The statistical test(s) used AND whether they are one- or two-sided<br>*Only common tests should be described solely by name; describe more complex techniques in the Methods section.*
☐	☒	A description of all covariates tested
☐	☒	A description of any assumptions or corrections, such as tests of normality and adjustment for multiple comparisons
☐	☒	A full description of the statistical parameters including central tendency (e.g. means) or other basic estimates (e.g. regression coefficient) AND variation (e.g. standard deviation) or associated estimates of uncertainty (e.g. confidence intervals)
☐	☒	For null hypothesis testing, the test statistic (e.g. *F*, *t*, *r*) with confidence intervals, effect sizes, degrees of freedom and *P* value noted<br>*Give P values as exact values whenever suitable.*
☒	☐	For Bayesian analysis, information on the choice of priors and Markov chain Monte Carlo settings
☒	☐	For hierarchical and complex designs, identification of the appropriate level for tests and full reporting of outcomes
☒	☐	Estimates of effect sizes (e.g. Cohen's *d*, Pearson's *r*), indicating how they were calculated

Our web collection on statistics for biologists contains articles on many of the points above.

Software and code

Policy information about availability of computer code

Data collection	L'Image (http://limagesoftware.net, August 2021), I-TASSER
Data analysis	hmmsearch v. 3.2.1, GraftM v. 0.11.1, FigTree v. 1.4.4, IQ-Tree v. 1.3.11.1, v. 1.6.8, v. 1.3.11.175, Cutadapt v.2.1.0, TrimGalore v.0.6.0, BBMap v. 38.85, metaSPAdes v. 3.14.0, v. 3.9.0, SAMtools v.1.3.1, bowtie2 v.2.2.9, MEGAHIT v.1.1.1-2-g02102e1, Trimmomatic v.0.36, MetaBAT2 v. 0.32.4, v. 1.7, MaxBin2 v. 2.2.4, metaQUAST v. 5.0.2, SPAdes v. 3.14.1, metaFLYE v. 2.8.2, CheckM v. 1.0.18-1.1.3, GTDB Toolkit v. 1.1.0, v. 1.4.1, Prodigal v. 2.6.2, eggNOG-mapper v. 2, ProtTest v. 3.4, FASconCAT-G v. 1.0.4, RAxML v. 8.2.12, COUNT v. 9.1106, DaliLite v. 5, JMP Pro v. 13.1.0

For manuscripts utilizing custom algorithms or software that are central to the research but not yet described in published literature, software must be made available to editors and reviewers. We strongly encourage code deposition in a community repository (e.g. GitHub). See the Nature Portfolio guidelines for submitting code & software for further information.

Data

Policy information about availability of data

All manuscripts must include a data availability statement. This statement should provide the following information, where applicable:

- Accession codes, unique identifiers, or web links for publicly available datasets
- A description of any restrictions on data availability
- For clinical datasets or third party data, please ensure that the statement adheres to our policy

Sequence data for nomenclatural types and some non-type genomes were deposited on GenBank under accession numbers listed in Supplementary Table 6, and all other genomes were submitted to eLMSG and/or IMG under under Bioproject no. PRJNA495050, PRJNA791658, PRJNA807863, PRJNA381623, and PRJNA780009, as listed in Supplementary Table 6. For nomenclatural types, raw sequence reads were submitted to the Sequence Read Archive (SRA) under the SRA run accessions

listed in Supplementary Table 6. All other data are contained within the manuscript and supplements/source data file.

Field-specific reporting

Please select the one below that is the best fit for your research. If you are not sure, read the appropriate sections before making your selection.

☐ Life sciences ☐ Behavioural & social sciences ☒ Ecological, evolutionary & environmental sciences

For a reference copy of the document with all sections, see nature.com/documents/nr-reporting-summary-flat.pdf

Ecological, evolutionary & environmental sciences study design

All studies must disclose on these points even when the disclosure is negative.

Study description	We investigated effects of tungsten on microbial growth in novel anaerobic thermophiles and used microbiological and computational techniques to characterize the effects observed, as well as to assess the magnitude of tungsten dependence among relatives of the cultivated taxa.
Research sample	Anoxic hot spring sediment enriched in thermophilic microorganisms as representation for anaerobic thermophilic communities. Enriched microorganism taxa are provided in Figure 1a. Culture conditions: The thirteen different media and incubation conditions used are shown in Supplementary Table 1. All media were based on GBS salts synthetic medium, which contained per liter of 18.2 MΩ · cm water: 3 g NaCl, 0.3 g Na_2SO_4, 0.15 g KCl, 0.015 g $CaCl_2 \cdot 2H_2O$, 0.123 g $MgSO_4 \cdot 7H_2O$, and 5 mL of a trace mineral solution65. The trace mineral solution, based on ref. 66, contained per liter: 3.7 g Na_2-EDTA, 1.1 g $FeSO_4 \cdot 7H_2O$, 0.028 g $ZnSO_4 \cdot 7H_2O$, 0.098 g $MnCl_2 \cdot 4H_2O$, 0.031 g H_3BO_3, 0.024 g $CoCl_2 \cdot 6H_2O$, 0.017 g $CuCl_2 \cdot 2H_2O$, and 0.024 g $NaMoO_4 \cdot 2H_2O$.
Sampling strategy	In-situ enrichments were not replicated, samples for the 13C-labeling experiment were dublicated, and sample sizes comprised at least 3 replicates otherwise, as these are commonly used in microbiological work. Great Boiling Spring (GBS) water used in preparation of cultivation medium was sampled in 2013, 2016, 2017 and 2018, passed through a 0.2 µm filter, and stored in 20 L plastic containers in the laboratory without temperature control. In situ enrichments on ammonia-fiber explosion-treated corn stover (kindly provided by Bruce Dale, Michigan State University) in GBS were performed as described in Peacock et al.17. Samples were taken after approximately six months of incubation on 29 March 2016. At that time, the temperature of the water was 86.0 °C and the pH was 7.15, typical for the main source pool of GBS16. For sampling, a nylon bag containing corn stover was removed from the conical tube, the bag was cut open with a sterile scissors, and approximately half of the contents was immediately transferred to 10 mL of 0.2 µm filtered GBS water (previously sampled, sparged with N_2, reduced by addition of cysteine to 0.05 g/L, and sterilized by autoclaving) in a 25 mL Balch tube under a stream of N_2 gas applied through a 0.2 µm filter. The tube was sealed with a butyl rubber stopper, the headspace was flushed for 3 minutes with N_2 gas, and the tube was then shaken to disperse the corn stover. Previously prepared anaerobic media (50 mL in 160 mL serum vials sealed with butyl rubber stoppers; see below) were inoculated with 0.2 mL of the corn stover slurry using a sterile, N_2-flushed needle and syringe. The bottles were transported back to the laboratory at ambient temperature and were transferred to high-temperature incubators within 24 hours of sample collection. The remaining in situ corn stover enrichment was placed in 1.5 mL centrifuge tubes, frozen immediately and transported back to the laboratory on dry ice, and then stored at –80 °C for DNA extraction.
Data collection	J.D., M.M., C.G., L.G., C.S., C.V., M.Z., N.B., and F.R. conducted cultivation experiments, monitoring and quantitative PCR. S.B. and J.A.D. analyzed qPCR and 16S data. D.M. performed CARD-FISH. X.M., P.K.W., and J.P.-R. analyzed culture samples with nanoSIMS and S.B. and A.E.D. joined for the nanoSIMS data analysis/interpretation. M.P., J.-Y.J., D.R.C., L.M.K., E.S.J., E.S.B., A.D.C., B.S.C., Z.-S.H., W.-J.L., A.-L.R., and M.B.S. were involved in metagenomic sampling, sequencing, assembly, annotation and binning. M.P. performed phylogenomic analyses and ACS reconstructions. S.B. carried out the protein structural modeling.<br>W levels in laboratory media and filtered GBS spring water were measured using inductively-coupled plasma mass spectrometry (ICP-MS) after addition of nitric acid (OPTIMA grade, Fisher Chemical, Fair Lawn, NJ, USA) to 1 % final concentration at Huffman Hazen Laboratories (Golden, CO, USA). 16S rRNA gene tag amplification and sequencing on initial enrichment cultures was performed essentially as described68. Quantitative PCR assays were performed in 96 well plates (Applied Biosystems) in a StepOnePlus Quantitative Real-Time PCR machine (Applied Biosystems) using PowerUp SYBR Green Master Mix (Applied Biosystems). cDNA was amplified with primers targeting AigG4 predicted W-associated genes and AigG4 16S rRNA (Supplementary Table 5) in a Axygen® Maxygene™ II Thermal Cycler. Cells were visualized with a Leica DM5500B microscope with a 100 magnification dry immersion objective. Fluorescence and brightfield images were collected at diverse locations with FISH-positive cells and the X-Y location of those images were noted, as well as fiducial locations to enable navigation in the nanoSIMS. Well slides were cut with a diamond saw in order to fit on metal sample holders and analyzed on a CAMECA nanoSIMS 50 at Lawrence Livermore National Laboratory. For the AigG4 cultures established from the in situ enrichment at GBS, extracted DNA were sequenced using the Illumina MiSeq platform (2250 bp), along with Oxford Nanopore sequencing using a MinION device. All extracted metagenomic DNA from geothermal springs from Tengchong, China and Yellowstone National Park, USA, and the marine hydrothermal vent in the Western Pacific were sequenced on the Illumina NovaSeq, HiSeq3000, or HiSeq4000 platforms, in a paired-end or single-read configuration (Supplementary Note 2).
Timing and spatial scale	Timing was adjusted to growth patterns of the enrichment culture and to maximize differences between treatments. All sample time points are clearly indicated in the data figures.<br>Great Boiling Spring (GBS) water used in preparation of cultivation medium was sampled in 2013, 2016, 2017 and 2018. Samples were taken after approximately six months of incubation on 29 March 2016. Stable isotope labeling was performed on-site at GBS in July 2017. Laboratory cultures were kept at 80 °C and initially transferred to fresh media (1/100 volume) every two weeks for the first two transfers after inoculation with in situ enrichments and then every three weeks thereafter.
Data exclusions	No data were excluded.

Reproducibility	When experimental steps were repeated (typically 2 experimental runs), resulting data were consistent with each other.
Randomization	Randomization is not relevant because microbiological samples taken contained a multitude of target species allowing statistical significance.
Blinding	Blinding is not relevant because organisms of interest cannot be visually identified.

Did the study involve field work? ☒ Yes ☐ No

Field work, collection and transport

Field conditions	Sunny, dry, 20-35 degree Celsius and hotter temperatures in close proximity to the spring.
Location	Great Boiling Spring, Gerlach, Nevada, USA (N 40 deg 39.6890 min, W 119 deg 21.9680 min).
Access & import/export	Sampling site is located on private property owned by Dave Jamieson and verbal permission to conduct field work has been standing for over 15 years.
Disturbance	No disturbance was caused to the spring.

Reporting for specific materials, systems and methods

We require information from authors about some types of materials, experimental systems and methods used in many studies. Here, indicate whether each material, system or method listed is relevant to your study. If you are not sure if a list item applies to your research, read the appropriate section before selecting a response.

Materials & experimental systems

n/a	Involved in the study
☒	☐ Antibodies
☒	☐ Eukaryotic cell lines
☒	☐ Palaeontology and archaeology
☒	☐ Animals and other organisms
☒	☐ Human research participants
☒	☐ Clinical data
☒	☐ Dual use research of concern

Methods

n/a	Involved in the study
☒	☐ ChIP-seq
☒	☐ Flow cytometry
☒	☐ MRI-based neuroimaging
