# Supplementary material for: An essential role for tungsten in the ecology and evolution of a previously uncultivated lineage of anaerobic, thermophilic Archaea
Source: Nat Commun. 2022 Jun 30;13:3773. doi: 10.1038/s41467-022-31452-8 (PMC9246946; doi:10.1038/s41467-022-31452-8)
Supplement: Supplementary file 4 — Description of Additional Supplementary Files [file 41467_2022_31452_MOESM4_ESM.pdf]

Title: Supplementary Data 1.
Description: Trace element analysis on GBS spring water and lab media by ICP-MS. Performed at the Analytical Chemistry Laboratory of New Mexico Bureau of Geology and Mineral Resources, New Mexico Institute of Minings and Technology. Detection limits for GBS spring water are higher because a 1/20 dilution of the sample was used for analysis. (Note that ICP-MS and ICP-OES analyses focusing on tungsten were performed separately).

Title: Supplementary Data 2.
Description: Levels of tungsten in GBS spring water and lab media. Molarity was calculated using a molecular mass of 183.84 for tungsten.

Title: Supplementary Data 3.
Description: Information on genome sequences used in this study.

Title: Supplementary Data 4.
Description: Average Amino Acid and Nucleotide Identities. a, Average Nucleotide Identity values (ANI) among members of Ca. Geocrenenecus. b, Average Nucleotide Identity values (ANI) among members of Ca. Terraquivivens. c, Average Nucleotide Identity values (ANI) among members of Ca. Wolframiiraptor. d, Average Amino Acid Identity values (AAI) among members of Ca. Geocrenenecus. e, Average Amino Acid Identity values (AAI) among members of Ca. Terraquivivens. f, Average Amino Acid Identity values (AAI) among members of Ca. Wolframiiraptor.

Title: Supplementary Data 5.
Description: Summary of GraftM output.

Title: Supplementary Data 6.
Description: COUNT session file for ancestral character state reconstruction.

Title: Supplementary Data 7.
Description: Tungsten-dependent ferredoxin oxidoreductase phylogeny.

Title: Supplementary Data 8.
Description: Single-gene phylogenies for physiology-related genes.

Title: Supplementary Data 9.
Description: Normalized ASV counts. All the samples were normalized to the minimum number of 5739 reads using SRS (Beule L, Karlovsky P. 2020. Improved normalization of species count data in ecology by scaling with ranked subsampling (SRS): application to microbial communities. PeerJ 8:e9593 https://doi.org/10.7717/peerj.9593). The sampling depth 5739 was also verified by a rarefaction curve.
